# The relationship between road traffic collision dynamics and traumatic brain injury pathology

**DOI:** 10.1093/braincomms/fcac033

**Published:** 2022-02-12

**Authors:** Claire E. Baker, Phil Martin, Mark H. Wilson, Mazdak Ghajari, David J. Sharp

**Affiliations:** 1 Centre for Neurotechnology, Imperial College London, South Kensington Campus, London SW7 2AZ, UK; 2 HEAD Lab, Dyson School of Design Engineering, Imperial College London, South Kensington Campus, SW7 2AZ, UK; 3 TRL, Crowthorne House, Nine Mile Ride, Wokingham, Berkshire, RG40 3GA, UK; 4 Imperial College London Saint Mary Campus, St Mary’s Hospital, Praed Street, London W2 1NY, UK; 5 Department of Brain Sciences, Imperial College London, 86 Wood Lane, W12 0BZ, UK; 6 UK Dementia Research Institute, Care Research & Technology Centre, Sir Michael Uren Hub, Imperial College London, 86 Wood Lane, London W12 0BZ, UK

**Keywords:** injury biomechanics, traumatic brain injury risk, delta-*V*, road traffic collision dynamics, automatic collision notification emergency response

## Abstract

Road traffic collisions are a major cause of traumatic brain injury. However, the relationship between road traffic collision dynamics and traumatic brain injury risk for different road users is unknown. We investigated 2065 collisions from Great Britain’s Road Accident In-depth Studies collision database involving 5374 subjects (2013–20). Five hundred and ninety-five subjects sustained a traumatic brain injury (20.2% of 2940 casualties), including 315 moderate–severe and 133 mild–probable injuries. Key pathologies included skull fracture (179, 31.9%), subarachnoid haemorrhage (171, 30.5%), focal brain injury (168, 29.9%) and subdural haematoma (96, 17.1%). These results were extended nationally using >1 000 000 police-reported collision casualties. Extrapolating from the in-depth data we estimate that there are ∼20 000 traumatic brain injury casualties (∼5000 moderate–severe) annually on Great Britain’s roads, accounting for severity differences. Detailed collision investigation allows vehicle collision dynamics to be understood and the change in velocity (known as delta-*V*) to be estimated for a subset of in-depth collision data. Higher delta-*V* increased the risk of moderate–severe brain injury for all road users. The four key pathologies were not observed below 8 km/h delta-*V* for pedestrians/cyclists and 19 km/h delta-*V* for car occupants (higher delta-*V* threshold for focal injury in both groups). Traumatic brain injury risk depended on road user type, delta-*V* and impact direction. Accounting for delta-*V*, pedestrians/cyclists had a 6-times higher likelihood of moderate–severe brain injury than car occupants. Wearing a cycle helmet during a collision was protective against overall and mild-to-moderate-to-severe brain injury, particularly skull fracture and subdural haematoma. Cycle helmet protection was not due to travel or impact speed differences between helmeted and non-helmeted cyclist groups. We additionally examined the influence of the delta-*V* direction. Car occupants exposed to a higher lateral delta-*V* component had a greater prevalence of moderate–severe brain injury, particularly subarachnoid haemorrhage. Multivariate logistic regression models created using total delta-*V* value and whether lateral delta-*V* was dominant had the best prediction capabilities (area under the receiver operator curve as high as 0.95). Collision notification systems are routinely fitted in new cars. These record delta-*V* and automatically alert emergency services to a collision in real-time. These risk relationships could, therefore, inform how routinely fitted automatic collision notification systems alert the emergency services to collisions with a high brain injury risk. Early notification of high-risk scenarios would enable quicker activation of the highest level of emergency service response. Identifying those that require neurosurgical care and ensuring they are transported directly to a centre with neuro-specialist provisions could improve patient outcomes.

## Teaser

Baker *et al*. report that brain injury risk relates to road user vulnerability and collision dynamics. Pedestrians and cyclists were at the greatest risk. High lateral change in velocity exposure increased car occupant brain injury risk. Cycle helmet protection was not due to travel or impact speed differences between helmeted and non-helmeted cyclists.

## Graphical Abstract

**Graphical Abstract:**
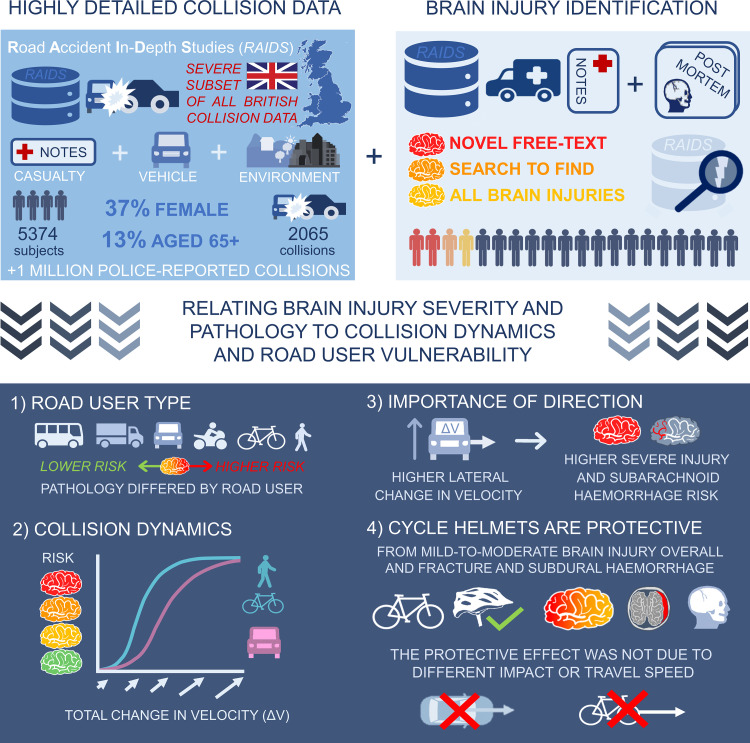

## Introduction

Each year, 1.35 million people are killed in road traffic collisions (RTCs) globally, with at least 50 million people surviving after sustaining injuries.^1^ Traumatic brain injury (TBI) is a leading cause of death and disability following RTCs, with an estimated 34 million people sustaining TBI in RTCs globally each year.^2^ Almost 70% of all RTC fatalities involve a head injury, with 32% due to isolated head injuries.^3^ In Europe, RTCs are the commonest cause of severe TBI.^4–6^ The majority of those injured are ‘active adults’ aged 16–55 years. This produces major long-term socioeconomic impacts, with TBI estimated to cost the global economy approximately $US400 billion annually.^7^

RTCs commonly lead to a range of TBI pathologies. The type of injury relates to the RTC dynamics.^8,9^ However, despite the global impact of TBI, there is limited understanding of this relationship. This is a key knowledge gap because reducing the risks associated with RTCs depends on an understanding of how forces produced during a collision cause TBI. A large in-depth database has been developed in Great Britain (GB) in recent years that provides detailed information about dynamics from real-world collisions as well as information about any TBI sustained. The Road Accident In-Depth Studies (RAIDS) database is collected on behalf of the UK Government’s Department for Transport with the aim of reducing serious injuries and fatalities on British roads.^10^ It contains information about both the collision scenario (including vehicle dynamics) and clinical information from hospital records and post-mortem reports. The availability of this data allows a detailed investigation of the risk of TBI associated with specific types of collision in different types of road users, including those more vulnerable to injury such as cyclists and pedestrians.

RTC reconstruction enables the vehicle dynamics and biomechanics of vulnerable road users (VRUs) involved in a collision to be estimated from evidence collected after the event, such as scene photographs, CCTV or dashcam footage and vehicle damage profiles.^11^ This information provides the opportunity to investigate the causation of TBI. The total change in velocity during the impact phase of each vehicle involved in a collision (delta-*V*) is a key measure. This can be calculated retrospectively and is known to predict overall injury severity.^12,13^ Delta-*V* provides an indication of the change in kinetic energy a vehicle is exposed to during a collision, some of which is transferred to the occupants causing injury. Total delta-*V* takes into account both lateral (side-to-side) and longitudinal (front-to-back) delta-*V*, with this directionality influencing injury risk.^14^

Severe TBI is more common in car occupants involved in side-impact collisions, which are dominated by lateral delta-*V*.^14^ Previous work has often used compound measures of injury severity such as the abbreviated injury scale (AIS).^15–17^ This approach limits the ability to investigate the causation of different TBI pathologies as it can be difficult to accurately obtain information about the underlying TBI pathology from the AIS region severity score recorded in databases and some types of TBI can be omitted from the individual AIS injury codes.^18–20^ A small amount of work has focused on the relationship between specific TBI pathologies and RTC dynamics. Two studies showed that collision dynamics (including delta-*V*) correlate with the volume of subdural and intraventricular haemorrhage.^21,22^ RAIDS allows us to extend this work by providing detailed information about the nature of TBI pathology both from clinical records and post-mortem reports of more than 5000 subjects involved in over 2000 collisions. This allows the specific investigation into the relationship between RTC dynamics and TBI pathologies including subdural haematoma (SDH), subarachnoid haemorrhage (SAH), extradural haematoma, focal brain injuries and diffuse axonal injury (DAI).

The effects of collisions are different for different types of road users. VRUs constitute significant numbers of the overall casualties, with motorcyclists making up 24% of all casualties in one study, and pedestrians and cyclists 17% each.^23^ There is a higher risk of TBI in VRUs which includes pedestrians, cyclists and motorcyclists. For example, one European study showed increased odds ratios for severe TBI compared with restrained car occupants of 18.1 for non-helmeted motorcyclists, 9.2 for pedestrians, 3.9 for unrestrained car occupants and 2.8 for helmeted motorcyclists.^24^ One study showed a relationship between TBI and vehicle impact speed in pedestrians and cyclists.^25^ Another showed that cyclists most commonly sustained serious TBI with loss of consciousness (LOC) and base of skull fractures.^26^ However, there has been little systematic work relating vehicle dynamics and impact biomechanics to TBI in different types of road users, despite the obvious differences in exposures between restrained vehicle occupants and VRUs.

Here, we use the RAIDS database to study 5374 subjects involved in GB injury-causing RTCs between 1 April 2013 and 31 March 2020. Our work provides the first description of TBI prevalence from RAIDS data. We scale to the national (GB) level using data of >1 000 000 police-recorded RTC casualties to provide the first GB-wide estimates of TBI pathology prevalence and risk for different road users due to RTCs derived from collision data. The change in velocity (delta-*V*) calculated for the vehicles involved in each RAIDS collision is then related to the risk of sustaining different types of TBI. A free-text search algorithm we developed enabled us to extract information directly from the detailed injury descriptions, ambulance notes, clinical reports and post-mortem information that would have been inaccessible using AIS injury codes alone, enabling a more complete analysis of the data. For >5000 casualties involved in collisions, we (i) estimated TBI severity (using the Mayo classification system) and identified TBI pathologies; (ii) calculated the prevalence of TBI severity and pathologies in RAIDS and scaled these results to the GB population; (iii) investigated vehicle dynamics and biomechanical descriptions of the collisions for different road users and (iv) investigated the relationship between delta-*V* and TBI, producing injury risk curves for car occupants and a combined pedestrian–cyclist road user group.

## Methods

### In-depth collision data collection

RAIDS data collection is a collaborative effort between the police, hospitals and dedicated on-site investigation units from Loughborough University and the Transport Research Laboratory (TRL). All cases have >3000 fields detailing the casualty’s injuries, vehicle information, collision causation and environmental factors. Injury information comes from clinical records (including ambulance notes, hospital records and any radiology and post-mortem text available). There are two types of RAIDS collision investigations: on-scene and retrospective investigations. All investigations use collision reports and photos are received from dedicated police Collision Investigation Units. On-scene investigations are additionally attended by TRL’s dedicated collision investigation team. Further information can be found in Supplementary Table 1.

### Study in-depth data characteristics and inclusion criteria

We selected data from a 7-year period from 1 April 2013 to 31 March 2020. This includes collection Phases 1, 2 and 2 (Extension) (see Supplementary Table 1 for details). Our sample included 5374 subjects involved in 2065 collisions and 2940 casualties. Of the 5112 subjects with known gender information, 37% were female. Of the 4807 with known ages, 79% were 16–64 years, 13% were >64 years and 8% were children <16 years old. The casualty group included 252 fatalities, of which 227 (90%) had post-mortem information available. Clinical information sources included ambulance notes, hospital notes, patient questionnaires and radiology reports. Three hundred and eighty-eight less seriously injured subjects additionally returned self-report questionnaires. Where relevant to the collision event, pre-existing medical conditions were also known. The primary purpose of the RAIDS database is to determine how and why serious injuries and fatalities are occurring on the roads, to mitigate against them. It is important to note that the collisions included in the database tend to be more serious. A full breakdown of the inclusion criteria is shown in Supplementary Table 1.

### Traumatic brain injury classification

**Figure 1:**
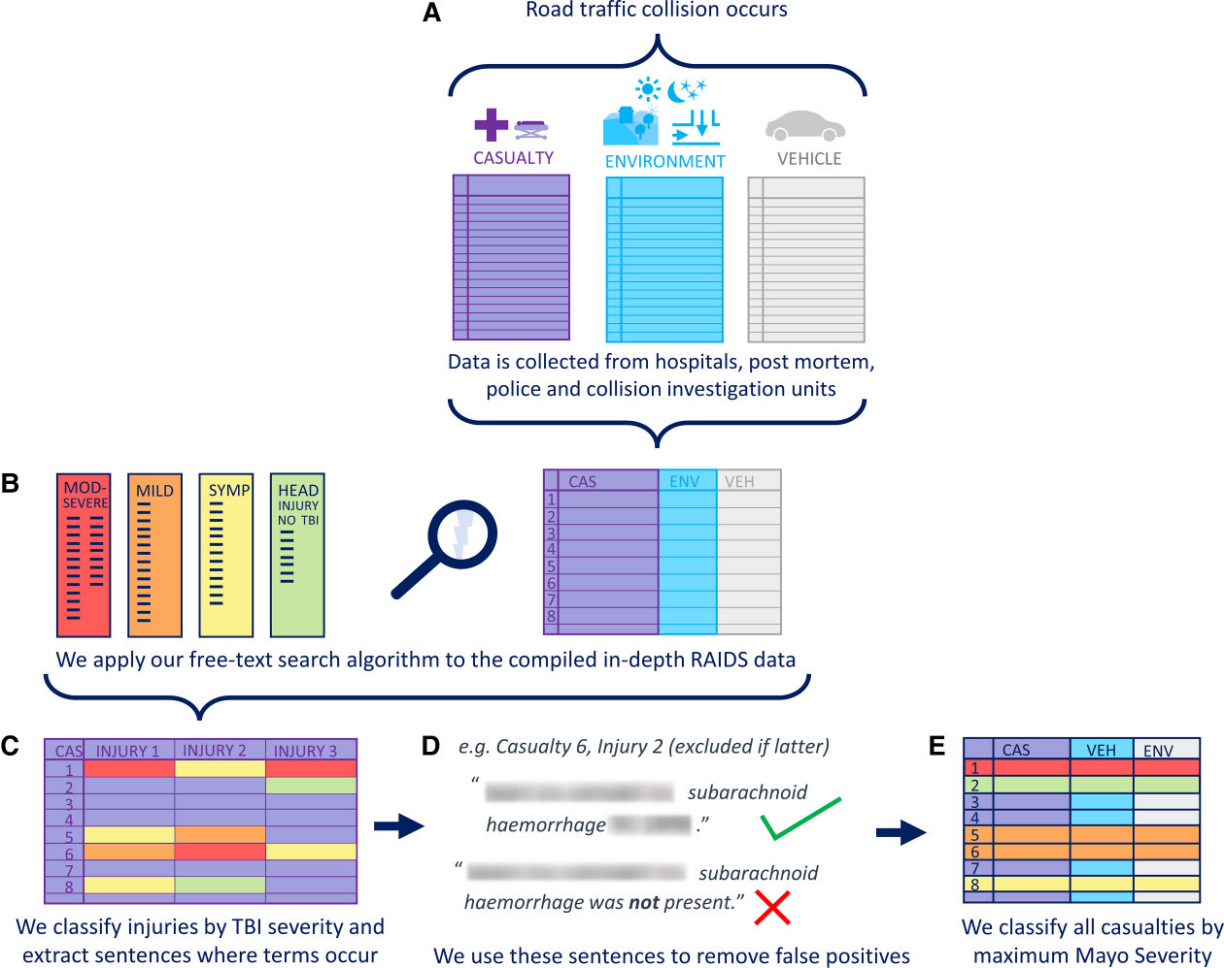
**Free-text search algorithm visual summary**. Free-text search algorithm visual summary showing the classification of TBI from the RAIDS dataset. (**A**) RAIDS data are collected and compiled; (**B**) free-text search algorithm is used to identify relevant information from all text recorded in RAIDS; (**C**) TBI severity and pathology present for each subject are found by recording terms found in the database and extracting the relevant sentence for context; (**D**) the extracted sentences are used to identify and remove false positives; (**E**) each casualty is given a final maximum TBI severity label from their injuries.

RAIDS uniquely captures detailed clinical and collision information, making our analysis of how RTC dynamics relate to TBI severity and pathology possible. However, as RAIDS is not primarily intended for TBI research, certain data elements that are commonplace in large studies designed specifically for TBI research are not available. Therefore, we extracted information using the free-text search algorithm was used to estimate the TBI severity using the Mayo Clinic Classification^27^ (Fig. 1). The Mayo system combines several TBI indicators including the Glasgow coma scale (GCS), LOC, post-traumatic amnesia and the presence of specific pathology, including brain haemorrhages, contusions and skull fracture for classification. The Mayo system incorporates clinical and neuroimaging information with GCS and LOC, allowing for the most comprehensive classification with the data we had available to us. GCS was known for 1725 subjects (62.4% of those injured), LOC on arrival was known for 1783 subjects (62.4% of those injured) and neuroimaging information was available for at least 398 RAIDS subjects.

### Free-text search algorithm

We developed a free-text search algorithm in Python that extracted TBI information using regular expression search patterns. The search terms related to TBI pathology, symptoms and treatments selected by the authors and reviewed by an expert histopathologist and TBI clinician can be found in Supplementary Table 2. The search terms were refined using RAIDS Phase 1 and Phase 2 data (2013–19) and validated manually for 507 subjects involved in 200 collisions from Phase 2 Extension data (2019–20) obtaining ≥99.4% agreement. Our method also captured all AIS injury-coded pathologies it was possible to directly compare (SDH, SAH and skull fracture). We accounted for misspelling and acronyms and extracted the sentence the term appeared in to enable false positives to be removed (e.g. where ‘no’ preceded a search term) before classifying each subject by overall Mayo severity.

### Scaling TBI severity and pathology in RAIDS to the police-reported GB collisions

The RAIDS database contains subjects who are generally more severely injured and is, therefore, not a representative subset of all GB collisions. The most comprehensive GB RTC database, STATS19, does not contain specific injury information.^28^ Therefore, to estimate TBI prevalence in police-reported collisions nationally (2013–19), we use both RAIDS and STATS19 scale our findings from RAIDS using 1 102 567 police-reported RTC casualties (12 881 fatalities, 152 788 serious injuries and 951 923 slight injuries). We use seven fields present in both RAIDS and STATS19 (road user type, casualty age, lighting level, speed limit, road class and vehicle age and overall injury severity) to calculate the scaling weights. We use *χ*^2^-testing to confirm that each variable distribution differs significantly between datasets. The ‘rpart’ recursive partitioning R package decision trees were used to select which one or two fields which best predict overall injury severity.^29^ Cases in each dataset are clustered by subcategories (e.g. an age group and road user type) and the cluster proportion of the dataset as a whole is calculated. A mapping is created between the corresponding cluster proportions. Our methodology is similar to other scaling methods between in-depth and national sources, refined by TRL statisticians to fit the nuances of GB data.^30^ A full description of the scaling method is given in the Supplementary material along with calculated weights used for the scaling (Supplementary Tables 3–6).

### Retrospective delta-*V* calculation

**Figure 2:**
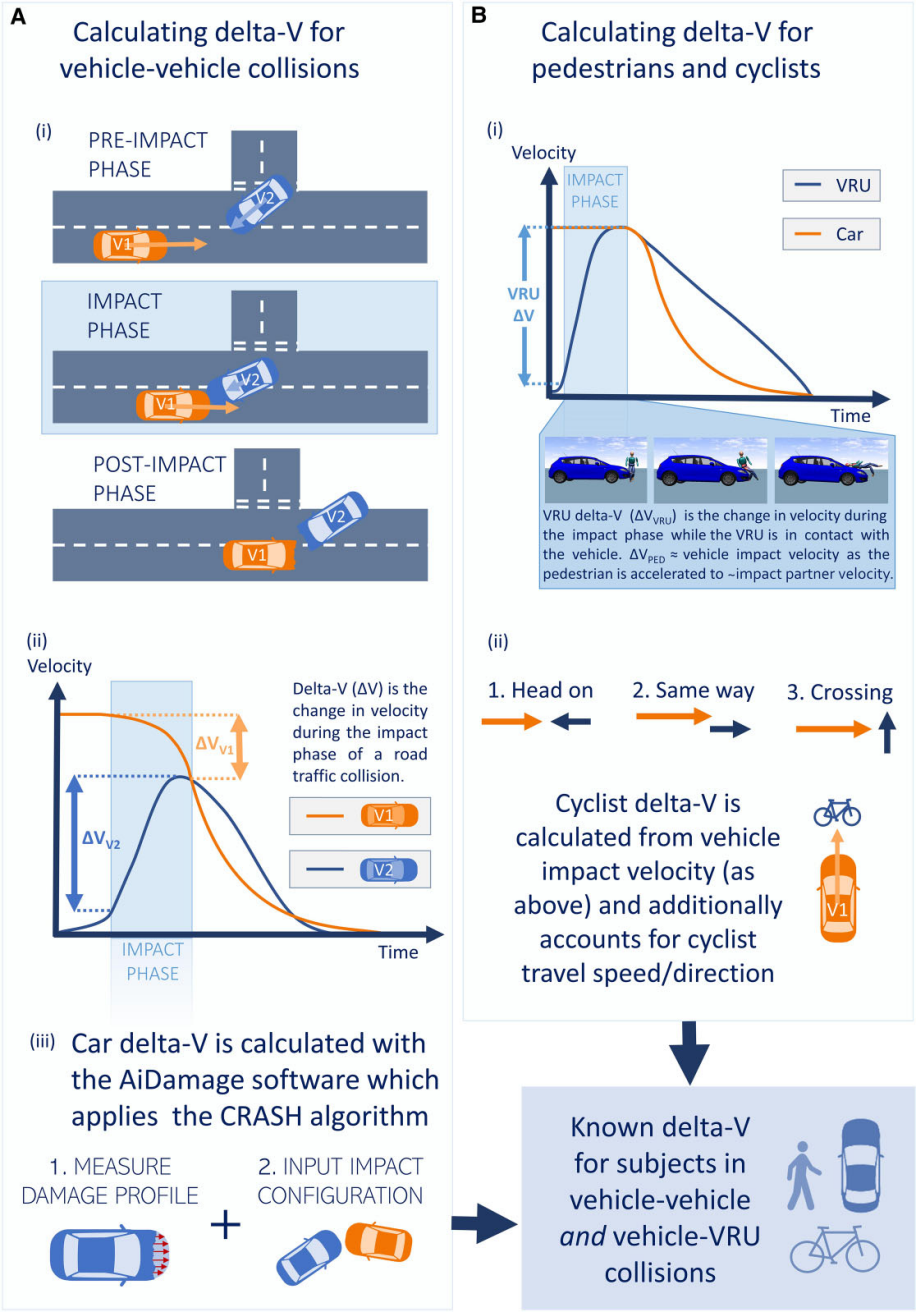
**Calculating delta-*V* for different road users**. Calculating delta-*V* for different road users. RAIDS collisions (*n* = 2065) include: (**A**) vehicle-to-vehicle and (**B**) vehicle-to-vulnerable road user (VRU i.e. cyclists and pedestrians) collisions. Delta-*V* calculation differs for **A** and **B** due to the differences in collision dynamics. **A**(i) shows the three main phases of vehicle-to-vehicle collisions. **A**(ii) shows example vehicle velocities for two vehicles (V1 and V2) during a collision. The distinct delta-*V* for each vehicle (Δ*V*_V1_ and Δ*V*_V2_) correspond to the change in velocity during the impact phase. **A**(iii) shows how delta-*V* is calculated retrospectively from crush measurements and vehicle trajectories using the AiDamage programme and CRASH algorithm. **B**(i) illustrates the VRU delta-*V* corresponding to the impact phase as the VRU is accelerated up to the speed of the other vehicle involved in the collision. **B**(ii) shows how the other vehicle’s velocity is used in conjunction with VRU velocity to calculate the relative delta-*V* between the VRU and vehicle involved.

The detailed collision information recorded in RAIDS specifically enables metrics describing vehicle dynamics and VRU biomechanics to be calculated even when these are not recorded by the vehicle during the collision. Collisions are split into three main phases: pre-crash, impact and post-crash (Fig. 2Ai). Delta-*V* is calculated during the impact phase i.e. from the moment of impact to the moment of separation, providing a measure of the change in velocity of a vehicle or VRU during the impact phase (Fig. 2Aii and Bii). Delta-*V* relates to overall injury severity.^14,31,32^ Delta-*V* in this study is of the vehicle or VRU overall, not the delta-*V* local body region (e.g. head), which can vary based on the specific kinematics of the collision. Broad collision dynamics are different for vehicles and VRUs, so delta-*V* was calculated differently. Vehicle delta-*V* is determined by RAIDS investigators from crush profile measurements and (where available) initial trajectories (Fig. 2A). The AiDamage programme is used to reconstruct the collision from this information, applying the computer reconstruction of automobile speeds on the highway (CRASH) algorithm to determine energy-related parameters including delta-*V*.^33,34^ Longitudinal (front-to-back), lateral (side-to-side) and total delta-*V* are calculated for each vehicle (Fig. 2Aii). Delta-*V* calculated using CRASH3 accurately reflects in-vehicle sensor measurements, particularly for car-to-car impacts (to within 2 km/h), which make up the majority of cases.^35^ Small differences (mean absolute error −4 km/h) have been shown to exist between CRASH3 delta-*V* and event data recorder (EDR) in-vehicle sensor measurements of delta-*V* in European vehicles. We chose not to apply a correction to account for this small discrepancy because the precise relationship to the fleet represented in our dataset is unknown.

Occupants in all cars with valid delta-*V* estimates from single-impact phases were included. Where multiple impacts were present, delta-*V* was included only if one of the impact phases was clearly injury-causing. Pedestrian delta-*V* is approximated as the impact speed of the vehicle because most pedestrians move slowly and were injured while crossing (no velocity component parallel to vehicle velocity). Cyclists travel at higher speeds, sharing the carriageway with vehicles. Therefore, their initial speed and direction are influential and taken into account (Fig. 2B). The parallel component of cyclist velocity is combined with the vehicle impact speed (Δ*V*_VRU_ = *V*_car initial_ + *V*_VRU initial_). This method assumes the VRU is accelerated to the speed of the impacting vehicle, hence VRUs directly runover (e.g. those already lying in the road prior to impact) were excluded as this assumption was not upheld. Further details on delta-*V* calculation can be found in the Supplementary material.

### TBI prevalence and relative risk calculation for different road users

Relative risk (RR) was used to estimate the risk of TBI pathologies and severities for different road user groups in police-reported GB RTCs. RR was calculated by dividing the rate in the exposed group *a*/*A* by the rate in the unexposed (or less exposed) group *b*/*B*,

$$\mathrm{RR}=\;\frac{a/A}{b/B}$$

where *a* and *b* are the number who sustained a given pathology or severity in the exposed and unexposed groups, respectively, and *A* and *B* are the total number in the exposed and unexposed groups, respectively.^36^ A 95% confidence interval (CI) on the RR was calculated using:

$$RR_{95\text{\%}\mathrm{CI}}=\mathrm{RR}\pm \exp(\ln(\mathrm{RR})\pm 1.96\times \mathrm{SE}(\ln(\mathrm{RR})))$$

where the standard error of log RR is given by

$$\mathrm{SE}(\ln(\mathrm{RR}))=\sqrt{1/a+1/b-1/A-1/B}$$

*χ*^2^-tests were used to determine statistically significant differences between pathology for different road users.^37^

### Analysis of delta-*V* severity and pathology using normalized cumulative distributions

Normalized cumulative frequency distributions were calculated and plotted in Python using Bokeh with 10 000 iterations used to produce bootstrapped 95% CIs.^38^ The relationship between delta-*V* distributions and TBI were analysed in groups with different TBI severities pathology compared to an injured group without TBI and an uninjured group. We determined 95% CIs using 10 000 bootstrap resamples and calculated 95% CIs from the 2.5th and 97.5th percentiles of the 10 000 ranked values at each point.^39,40^ Shapiro–Wilk normality testing showed that data in the majority of the pathology groups and TBI severity groups were not normally distributed. Bootstrapping does not rely on parametric statistics and is, therefore, well-suited to calculating CIs for our dataset. For cross-group analysis of the TBI pathology, we applied a one-sided Mann–Whitney (MW) U-test to determine whether delta-*V* distribution showed differences across groups.^41^

### Determining how dominant vehicle delta-*V* component direction affects TBI prevalence

We next examined the relationship between lateral and longitudinal delta-*V* and TBI. We first considered the groups exposed to delta-*V* dominated by the one component and then the groups of car occupants exposed to only lateral and only longitudinal delta-*V* components using *χ*^2^ and RR analysis. For non-normally distributed data, Kruskal–Wallis one-way ANOVA tests were used to compare delta-*V* distributions for different TBI groups. There is a potential confounding factor where higher TBI prevalence in one group could be caused by higher delta-*V* distribution in the delta-*V* component which dominates it. Hence, we applied a one-sided MW U-test to determine whether the lateral delta-*V* distribution is higher than the corresponding longitudinal delta-*V* distribution.^41^

### Logistic regression for the calculation of injury risk

We used binary logistic regression to produce injury risk curves for different road users and TBI pathologies using total delta-*V*.^42–44^ To further understand how RTC dynamics influence TBI in car occupants, we constructed a multivariate logistic regression model using a binary flag for dominant lateral delta-*V* and total delta-*V*. We additionally used multivariate logistic regression with the road user group and total delta-*V* as predictors to determine the odds ratio between road user groups. We ensured our sample sizes were large enough using the guideline *N* = 10*k*/*p* (*k* =  no. of covariate independent variables and *p* = smallest proportion of negative/positive cases in the population).^45^ The Python scikit-learn package was used to create the logistic regression model with limited-memory Broyden–Fletcher–Goldfarb–Shanno optimization and no regularization.^46^ Stratified *k*-fold cross-validation was used to create separate testing and training datasets, avoid overfitting to the training dataset and account for unbalanced groups. Average performance was calculated across the *k*-folds. *k* = 5 was chosen to ensure representative testing and training datasets. *k*-fold cross-validation was repeated 200 times with prespecified data seeds used to ensure repeatability when randomly shuffling the data prior to partitioning at the start of each iteration. The average risk and 95% CIs were determined from 1000 iterations using the 50th, 2.5th and 97.5th percentile ranked risk values at each point. To determine the predictive capability of our injury risk curves, we use the receiver operator characteristic (ROC) and associated area under the curve (AUC) averaged over all 1000 iterations. We provide further details on this method in the Supplementary material including the resulting precision and recall values (Supplementary Table 7).

### Statistical analysis

The application of key statistical techniques is summarized in this section. Statistical analyses applied within this manuscript are outlined in full in the previous method subsections, giving their context to the different sub-analyses. One-sided MW U-tests were used for determining whether there were statistically significant differences between delta-*V* distributions (both between different distributions in the same group and between the same distribution for different groups). *χ*^2^-tests were used to determine statistically significant differences between groups (e.g. pathology rates for different road users) and to test independence in the scaling methodology. We use the area under the ROC curve to compare logistic regression models. We calculate the standard error associated with the RR ratio. 95% CIs, test statistics and *P*-values are reported whenever possible.

### Ethical approval summary

RAIDS collection and use require very stringent ethical approvals and data security processes. These include approved applications for both a Confidentiality Advisory Group and the Research Ethics Committee and required the completion of a Data Security Protection Toolkit for ethical approval. To collect anonymized injury data, RAIDS has an agreement under Section 251. Section 251 of the NHS Act 2006 and the Regulations enable the common law of duty of confidentiality to be temporarily lifted so that confidential patient information can be transferred to an applicant without the discloser being in breach of the common law duty of confidentiality. Therefore, RAIDS does not seek permission from those who are injured. If a person would like their information removed, they are able to request this in writing. Approval to use the database for specific projects is granted by the Department for Transport. The AutoTRIAGE project approval includes all tasks that have been conducted for this publication. Additionally, all outputs, including this submitted manuscript, are checked thoroughly for anonymity and to ensure all protocols have been correctly followed prior to dissemination.

### Data availability

The data required for this study has restricted access and can be obtained with permission from the Department for Transport (contact: RAIDS@dft.gsi.gov.uk). The corresponding author is happy to be contacted and direct other researchers in the data used once this access is obtained. Derived data supporting the findings of this study are available from the corresponding author on reasonable request.

## Results

### TBI prevalence in the RAIDS database

**Figure 3:**
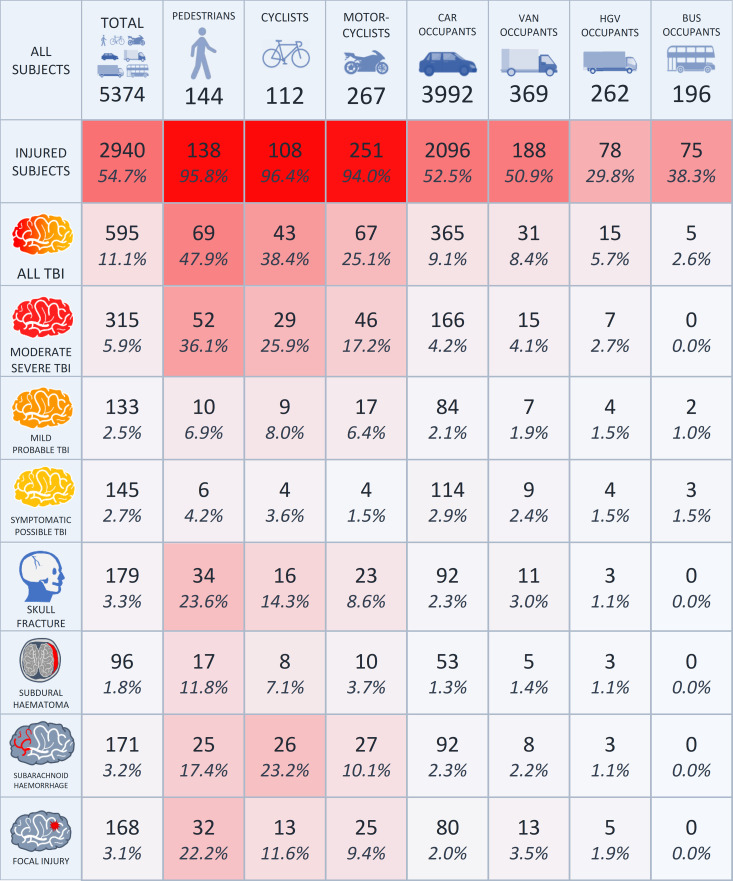
**Traumatic brain injury prevalence in RAIDS**. A summary of the TBI population in the RAIDS database. Numbers across all road user groups for a range of TBI severities and four most prevalent distinct pathologies are given with the corresponding percentage of all study subjects. In this figure, the percentages show the proportion of subjects in the column’s road user group that sustained the pathology in question. For example, there were 34 of 144 pedestrians who sustained a skull fracture, giving 23.6%. Thirty-two subjects of uncommon vehicle types are not given separate columns in this table but are included in the total count. Distinct groups of road users sustained different rates of TBI (*χ*^2^_(6)_ = 334.9, *P* < 0.001). The pedestrian, cyclist and motorcyclist vulnerable road user group had a higher prevalence of TBI compared with vehicle occupants (*χ*^2^_(1)_ = 279.1, *P* < 0.001).

Approximately half (47.9%, 1409 of 2940) of all casualties sustained an injury to the head and neck (AIS2005 region) (Fig. 3). Around 595 RAIDS subjects sustained TBI of any severity (20.2% of 2940 casualties, 11.1% of 5374 subjects). Of those with TBI, 315 (52.9%) were moderate–severe, 133 (22.4%) were mild–probable and 145 (24.4%) were symptomatic–possible. Distinct groups of road users sustained different rates of TBI (*χ*^2^_(6)_ = 334.9, *P* < 0.001). The pedestrian, cyclist and motorcyclist VRU group had a higher prevalence of TBI compared with vehicle occupants (*χ*^2^_(1)_ = 279.1, *P* < 0.001). They also had a higher prevalence of moderate–severe TBI (*χ*^2^_(1)_ = 398.1, *P* < 0.001). Fifty-two pedestrians (36.1% of 144 RAIDS pedestrians), 29 cyclists (25.9% of 112 RAIDS cyclists) and 46 motorcyclists (17.2% of 267 RAIDS motorcyclists) sustained moderate–severe TBI. In contrast, 166 car occupants (4.2% of 3992 RAIDS car occupants), 15 van occupants (4.1% of 369 RAIDS van occupants), 7 heavy goods vehicle occupants (2.7% of 262 RAIDS heavy goods vehicle occupants) sustained moderate–severe TBI.

The 595 RAIDS TBI casualties presented with a range of pathologies (Fig. 3). One hundred and seventy-one (3.2% of 5374 subjects) sustained a SAH. The prevalence of SAH differed across groups (*χ*^2^_(6)_ = 293.0, *P* < 0.001) and was most prevalent in the cyclist group. Around 23.2% of 112 RAIDS cyclists sustained an SAH followed by 17.4% of 144 RAIDS pedestrians and 10.1% of 267 RAIDS motorcyclists, compared with 2.3% of 3992 RAIDS car occupants. One hundred and sixty-eight (3.1% of 5374 subjects) sustained a focal brain injury. The prevalence of focal injury also differed across groups (*χ*^2^_(6)_ = 250.6, *P* < 0.001) and was more prevalent in pedestrians, cyclists and motorcyclists than in vehicle occupants (*χ*^2^_(1)_ = 194.5, *P* < 0.001). About 22.2% of 144 RAIDS pedestrians sustained a focal brain injury followed by 11.6% of 112 RAIDS cyclists and 9.4% of 267 RAIDS motorcyclists, compared with 2.0% of 3992 RAIDS car occupants. The focal injury was significantly higher for pedestrians than for two-wheeler road users (cyclists and motorcyclists) (*χ*^2^_(1)_ = 11.6, *P* < 0.001). One hundred and seventy-nine (3.3% of 5374 subjects) sustained a skull fracture, which was most prevalent in the pedestrian group (23.6% of 144 RAIDS pedestrians). Skull fracture prevalence was higher for the pedestrian, cyclist and motorcyclist VRU group than vehicle occupants (*χ*^2^_(1)_ = 195.8, *P* < 0.001) and higher for pedestrians than two-wheeler road users (cyclists and motorcyclists) (*χ*^2^_(1)_ = 13.3, *P* < 0.001). Ninety-six (1.8% of 5374 subjects) sustained an SDH, which was most prevalent in the pedestrian group (11.9% of 144 RAIDS pedestrians). SDH prevalence was higher for the pedestrian, cyclist and motorcyclist VRU group than vehicle occupants (*χ*^2^_(1)_ = 77.8, *P* < 0.001) and higher for pedestrians than two-wheeler road users (cyclists and motorcyclists) (*χ*^2^_(1)_ = 77.6, *P* < 0.001). Other less frequent pathologies included DAI (26, 0.5% of 5374 subjects) and extradural haemorrhage (16, 0.3% of 5374 subjects).

### The protective effect of cycle helmets during collisions

The vast majority of motorcyclists in our cohort wore helmets, in line with legal requirements. We, therefore, examined the cyclist population, which included a significant portion of non-helmeted cyclists. We considered the subset of 94 (84% of 112) cyclists with known helmet status. There was an almost exactly even split between those who wore a helmet (46, 49% of 94 cyclists) and those who did not (48, 51% of 94 cyclists). The prevalence of TBI of any severity was higher in the non-helmeted group (*χ*^2^_(1)_ = 6.84, *P* = 0.009). The prevalence of mild-to-moderate–severe TBI was also higher in the non-helmeted group (*χ*^2^_(1)_ = 5.15, *P* = 0.023), as well as the prevalence of skull fracture (Fisher exact *P* = 0.008) and SDH (Fisher exact *P* = 0.006). Two helmeted cyclists and 12 non-helmeted cyclists sustained a skull fracture. No helmeted cyclists sustained an SDH, compared with eight non-helmeted cyclists who did.

We investigated whether a difference in the impact and travel speeds between the helmeted and non-helmeted groups was contributing to the protective effect of helmets (e.g. non-helmeted cyclists being impacted by vehicles travelling at higher speeds or because they were cycling faster). Of the 94 cyclists with known helmet status, we, therefore, examined two additional subsets with known speed information. Among the 68 cyclists where both helmet status and the speed of the impacting vehicle were known, 37 non-helmeted (54% of 68) cyclists still showed a significantly higher prevalence of overall TBI, mild-to-moderate–severe TBI, skull fracture and SDH. Similarly, there was a higher prevalence of TBI among the 32 non-helmeted (55% of 58) cyclists with known travel speeds prior to the collision. In both subsets, there were no significant differences in the speed distributions between the helmeted and non-helmeted cyclist populations (cyclist travel speed: *U*_MW_ = 425.5, *P* = 0.443; vehicle impact speed: *U*_MW_ = 487.0, *P* = 0.859).

### TBI prevalence on GB’s roads

**Figure 4:**
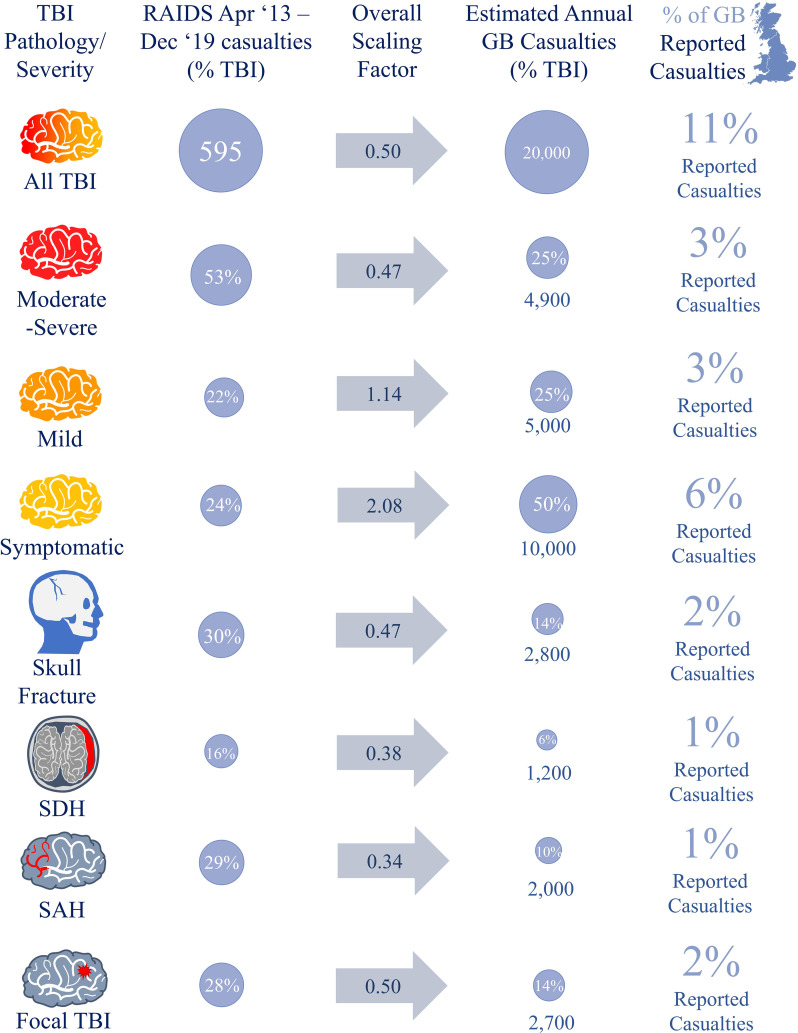
**Summary of RAIDS-STATS19 scaling results**. A summary of RAIDS-STATS19 scaling results showing the number of casualties sustaining TBI, split by severity and pathology, in RAIDS from 1 April 2013 to 31 December 2019 (excluding the 6-month period after the end of Phase 1 but before the start of Phase 2 collection in the first two quarters of 2016 when no RAIDS data were collected). The GB average estimated annual numbers during this period.

From 1 April 2013 to 31 December 2019, STATS19 recorded 1 190 717 police-reported casualties and 12 881 fatalities on GB’s roads (∼176 000 casualties annually). Excluding 6 months where no RAIDS data were collected, the remaining 75-month period included 1 102 567 STATS19 casualties. Extrapolating from our RAIDS findings we estimate that ∼20 000 (11% of ∼176 000 casualties) sustain a TBI each year: 4900 (24.6% of TBI casualties) moderate–severe, 5074 (25.4% of TBI casualties) mild–probable and 10 000 (50.0% of TBI casualties) symptomatic–possible (Fig. 4). Of the estimated 10 000 who sustain a mild–probable or moderate–severe TBI annually, we estimate 2800 (28.0%) sustain a skull fracture, 2700 (27.3%) sustain a focal brain injury, 2000 (20.5%) sustain an SAH and 1200 (12.1%) sustain an SDH.

### Relative risk of TBI for different road users in GB (scaled from RAIDS)

All VRUs had an increased risk of moderate–severe TBI compared with car occupants. Pedestrians, motorcyclists and cyclists (known to be underrepresented in STATS19) were 3.6, 2.7 and 1.3 times more likely to sustain a moderate–severe TBI than car occupants [RR_PED_ (CI_95%_): 3.65 (3.54–3.77); RR_MC_ (CI_95%_): 2.67 (2.58–2.77); RR_CYC_ (CI_95%_): 1.27 (1.21–1.33)]. Pedestrians were 5 times more likely to have focal brain injury [RR_FOCAL_ (CI_95%_): 5.35 (5.13–5.57)] and 3 times more likely to sustain a skull fracture and SAH [RR_SF_ (CI_95%_): 3.11 (2.99–3.24); RR_SAH_ (CI_95%_): 2.83 (2.70–2.97). Motorcyclists have a higher RR of focal brain injury and SAH than car occupants [RR_FOCAL_ (CI_95%_): 3.25 (3.08–3.41); RR_SAH_ (CI_95%_): 2.59 (2.45–2.73)].

### The relationship between total delta-*V* and moderate–severe TBI

**Figure 5:**
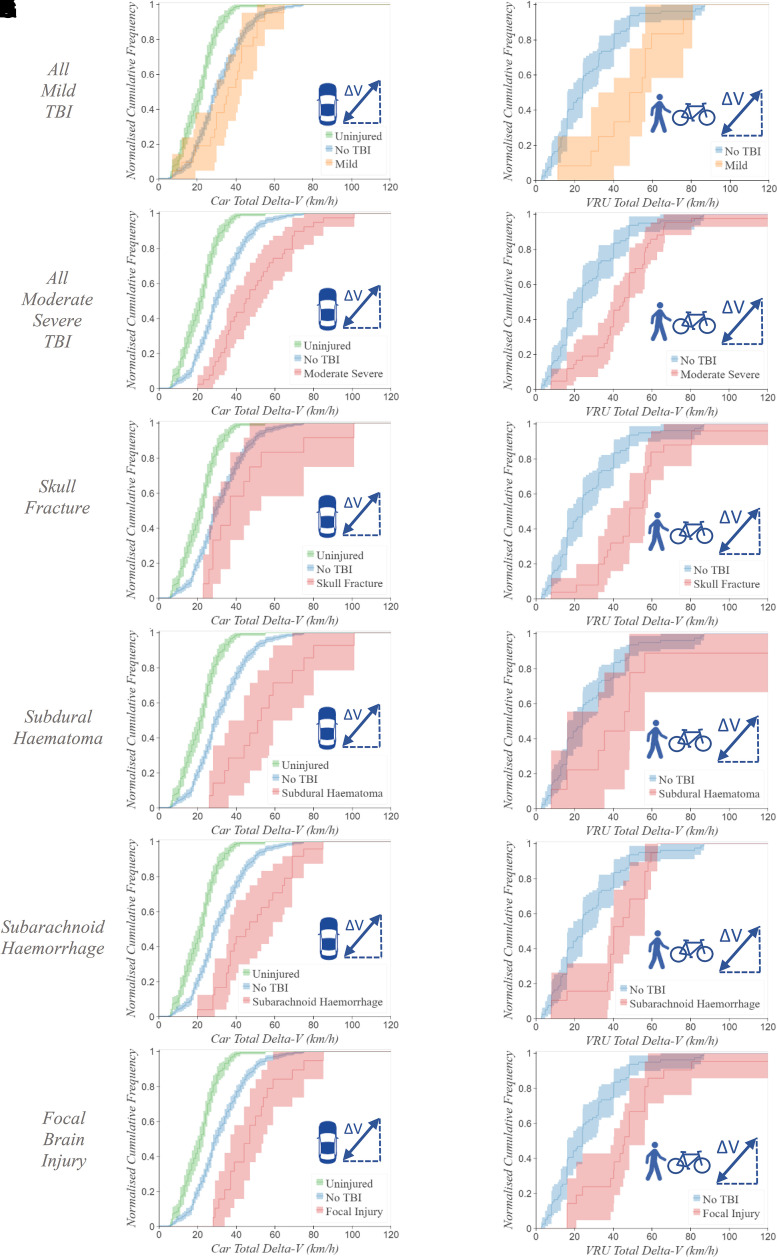
**Brain injury pathology normalized cumulative frequency distributions**. Normalized cumulative frequency distributions of total delta-*V* (km/h) are shown for TBI severity and a range of pathologies. The further to the right a curve is shifted, the higher the overall total delta-*V* distribution is. In **A** and **G**, the curve corresponding to mild TBI is shown in orange. In the remaining figures, the red curve shows moderate–severe TBI or key pathologies (labelled on the left-hand side of the figure). All uninjured car occupants (green curve, **A**–**F**) experienced delta-*V* ≤ 55 km/h and car occupant casualties without TBI (blue curve, **A**–**F**) experienced delta-*V* ≤ 75 km/h. In the pedestrian–cyclist group, there were insufficient numbers of uninjured subjects, so only casualties without TBI are shown (blue curves, **G**–**L**). The *y*-axis shows the proportion of the group which sustained their injury at or below the threshold on the *x*-axis. For example, 50% of car occupants with moderate–severe TBI were exposed to 45 km/h total delta-*V* or less. Corresponding shaded regions show 95% confidence intervals. Of 738 car occupants with known delta-*V*, 182 were uninjured, 472 were injured without TBI and 84 sustained TBI (24 symptomatic–possible, (**A**) 21 mild–probable and (**B**) 39 moderate–severe). Car occupants with known delta-*V* included (**C**) 14 with a skull fracture, (**D**) 14 with SDH, (**E**) 24 with SAH and (**F**) 19 with focal injury. Of 142 vulnerable road users with known kinematics, 3 were uninjured, 79 were injured without TBI and 60 sustained TBI (6 symptomatic–possible, (**G**) 12 mild–probable and (**H**) 42 moderate–severe). VRUs with known delta-*V* included (**I**) 25 with skull fracture, (**J**) 9 with SDH, (**K**) 19 with SAH and (**L**) 21 with focal injury.

We next examined the relationship between total delta-*V* and TBI severity for car occupants (*n* = 738) and a combined pedestrian–cyclist VRU group (*n* = 142) (Fig. 5). Car occupants who sustain moderate–severe TBI (*n* = 39) had higher total delta-*V* distributions than the uninjured (*n* = 182) (*U*_MW_ = 527.5, *P* < 0.001) and injured without TBI (*n* = 472) groups (*U*_MW_ = 4135.5, *P* < 0.001). No car occupants sustained moderate–severe TBI below 20 km/h total delta-*V* threshold. In contrast, 42% of uninjured car occupants were exposed to total delta-*V* below 20 km/h (Fig. 5B). The combined pedestrian–cyclist VRU moderate–severe TBI group (*n* = 42) also had higher total delta-*V* distributions than the injured without TBI (*n* = 79) group (*U*_MW_ = 778.0, *P* < 0.001). There were further differences in the thresholds above which specific TBI pathologies occur for car occupants (Fig. 5A–F) and the combined pedestrian–cyclist VRU group (Fig. 5G–L). Key pathologies examined were not observed below 19 km/h for car occupants (Fig. 5C–F) and below 8 km/h for VRUs (Fig. 5I–K). The focal brain injury had the higher threshold delta-*V* compared with the other pathologies for both car occupants (28 km/h, Fig. 5F) and VRUs (16 km/h, Fig. 5L). The four cyclists who sustained focal injury experienced total delta-*V* > 40 km/h. We did not have sufficient numbers within our sample to produce cumulative delta-*V* distributions for motorcyclists or vans and heavy goods vehicles.

### Lateral delta-*V* exposure increases car occupant TBI risk

**Figure 6:**
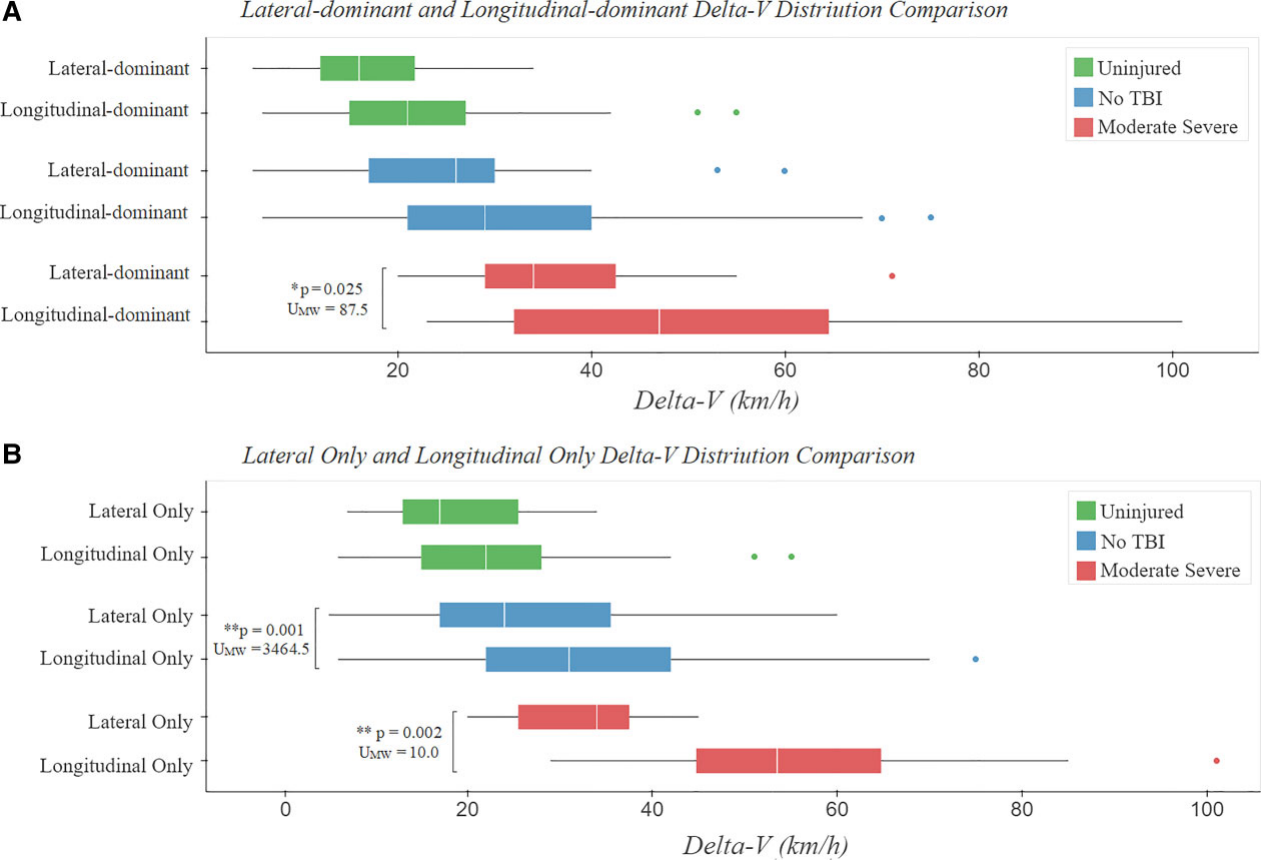
**Comparison of the lateral only and longitudinal only delta-*V* distributions.** Exploration of how the delta-*V* component direction affects TBI severity, comparing lateral and longitudinal delta-*V* distributions for car occupants. (**A**) shows the dominant delta-*V* component distributions for car occupants who experienced lateral-dominant delta-*V* and longitudinal-dominant delta-*V*. For moderate–severe TBI, the lateral-dominant delta-*V* distribution is significantly lower than the longitudinal-dominant delta-*V* distribution (*U*_MW_ = 87.5, *P* = 0.025), showing that the delta-*V* distribution is not a confounding factor. (**B**) shows the total delta-*V* distribution for car occupants who experienced only lateral and only longitudinal delta-*V*, split by TBI severity group. For moderate–severe TBI and casualties without TBI, the only lateral delta-*V* distribution is significantly lower than the only longitudinal delta-*V* distribution (injured without TBI, shown in blue): *U*_MW_ = 3464.5, *P* = 0.001; moderate–severe, shown in red: *U*_MW_ = 10.0, *P* = 0.002), showing that the delta-*V* distribution is not a confounding factor.

We next examined the effect of impact direction on TBI risk. Lateral and longitudinal delta-*V* was estimated for car occupants, where sufficient information was available (*n* = 738). Cumulative frequency curves for groups with moderate–severe TBI, injured subjects who did not sustain TBI and uninjured subjects were then calculated for lateral and longitudinal delta-*V* components (Fig. 6). Ten car occupants with equal lateral and longitudinal delta-*V* components were excluded. Car occupants involved in collisions with a higher lateral delta-*V* (*n* = 116) showed a higher prevalence of moderate–severe TBI than those with higher longitudinal delta-*V* (*n* = 614) (*χ*^2^_(1)_ = 5.36, *P* = 0.021) and 2.19 RR ratio, (CI_95%_:1.12–4.30). This difference was not driven by a higher delta-*V* distribution as the lateral-dominant group had lower total delta-*V* (*U*_MW_ = 28 815.5, *P* < 0.001) and lower dominant component distributions (*U*_MW_ = 2315.0, *P* < 0.001) compared with the longitudinal-dominant group (Fig. 6A).

A proportion of the collisions (37%, *n* = 270) involved both lateral and longitudinal delta-*V* components, so we performed a sub-analysis comparing collisions where car occupants were exposed only to lateral or longitudinal delta-*V*. Car occupants exposed only to lateral delta-*V* only (*n* = 61) had a higher risk of moderate–severe TBI than those exposed only to longitudinal delta-*V* groups (*n* = 407) [*χ*^2^_(1)_ = 7.99, *P* = 0.005; RR (CI_95%_): 3.34 (1.40–7.93)]. SAH was also more prevalent for car occupants only exposed to lateral delta-*V* (*χ*^2^_(1)_ = 5.41, *P* = 0.020) and had a 3.81 RR ratio (CI_95%_: 1.15–12.64). The lateral delta-*V* distributions were lower than the corresponding longitudinal delta-*V* distributions (Fig. 6B) (moderate–severe: *U*_MW_ = 10.0, *P* = 0.002; injured without TBI: *U*_MW_ = 3,464.5, *P* = 0.001, overall: *U*_MW_ = 9,811.0, *P* = 0.004).

### TBI risk increases with increasing total delta-*V* and road user vulnerability

**Figure 7:**
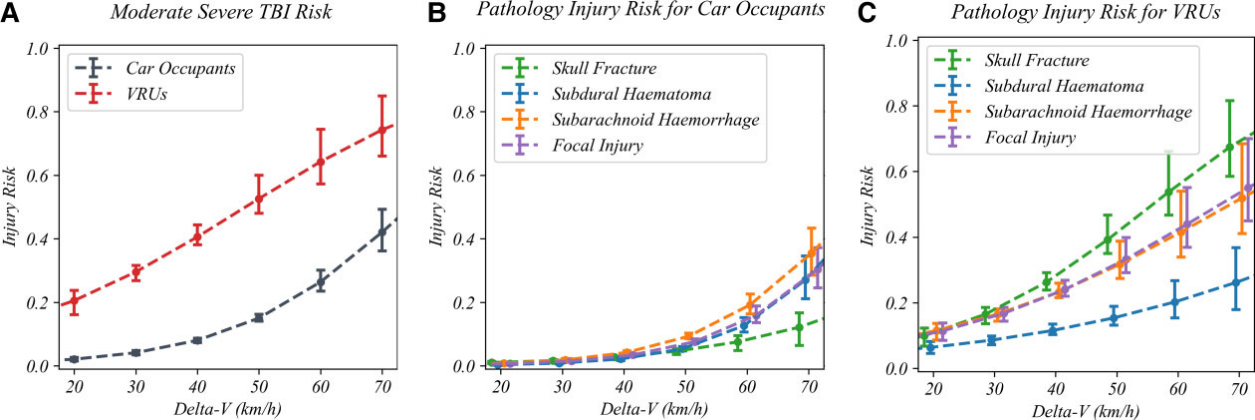
**Comparing vulnerable road user and car occupant brain injury risk.** A comparison of TBI risk for car occupants and the pedestrian–cyclist combined vulnerable road user group. The risk of all moderate–severe TBI at a particular delta-*V* value is higher for VRUs than for car occupants (*U*_MW_ = 8894.0, *P* < 0.001). (**A**). The risk of different TBI pathologies for car occupants (**B**) and VRUs (**C**) is shown for total delta-*V* values 20, 30, 40, 50, 60 and 70 km/h. For both VRUs and car occupants, the risk of subdural haematoma was lower than the other pathologies. For car occupants, subarachnoid haemorrhage was the highest risk pathology. For VRUs, focal injury, subarachnoid haemorrhage and skull fracture had similar risks. Individual comparisons between different road users for each of the four pathologies key can be found in Supplementary Fig. 2.

**Figure 8:**
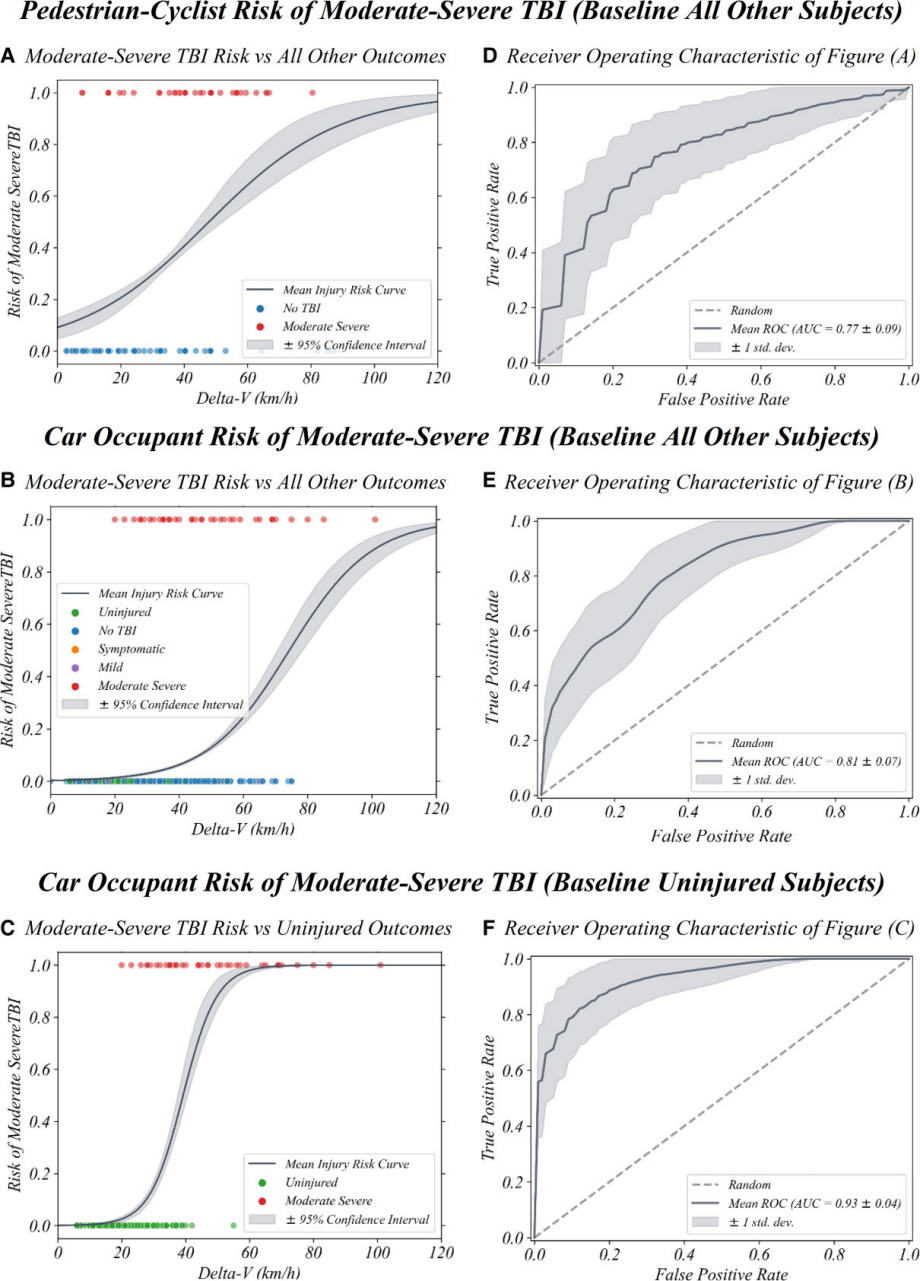
**Moderate–severe brain injury risk for different road users**. Moderate–severe TBI risk curves are shown for the combined pedestrian–cyclist VRU group [ROC_AUC_ (CI_95%_): 0.73 (0.55–0.89)] (**A**) and car occupants [ROC_AUC_ (CI_95%_): 0.81 (0.68–0.93)] (**B**) compared with all other subjects and (**C**) uninjured car occupants [ROC_AUC_ (CI_95%_): 0.93 (0.84–1.00)]. Corresponding ROC curves are also shown (**D–F**). Total delta-*V* (km/h) is the predictor used. Full logistic regression TBI pathology risk curves with delta-*V* as the predictor are shown for car occupants and VRUs in Supplementary Fig. 1.

Binary logistic regression was used to generate injury risk curves with delta-*V* as the predictor and TBI severity and pathologies as the outcome (Figs 7 and 8). The risk of sustaining a moderate–severe TBI was significantly higher for the combined pedestrian–cyclist VRU group than car occupants at all delta-*V*s (Fig. 7A) (*U*_MW_ = 8894.0, *P* < 0.001). Delta-*V* was also a significant predictor of outcome for all four most prevalent moderate–severe TBI pathologies (Fig. 7B and C) with the *P*-values associated with the *Z*-test for each pathology and road user group shown in Supplementary Fig. 1A–H.

Within the same road user group, there is significant overlap between the different TBI pathology risk curves which is unsurprising as numerous subjects sustained multiple TBI pathologies. For car occupants, SDH, SAH and focal injury are within 95% CIs of one another, with a lower risk of a skull fracture at a given delta-*V* (Fig. 7B). Contrastingly, skull fracture risk is greatest for VRUs, followed by SAH and focal injury which have similar risks (Fig. 7C). The odds ratios calculated from the coefficients of multivariate logistic regression with total delta-*V* and road user group as predictors (*Z*-test, *P* ≤ 0.002 in all instances) differed by TBI pathology when accounting for delta-*V* (*Z*-test, *P* ≤ 0.002 in all instances). For VRUs compared with car occupants, the odds ratio is higher for moderate–severe TBI [OR (CI_95%_): 6.84 (4.03–11.63)]. The difference was greatest for skull fracture [OR (CI_95%_): 12.32 (5.87–25.87)] (green lines on Fig. 7B and C), followed by focal injury [OR (CI_95%_): 8.34 (4.02–17.29)], SAH [OR (CI_95%_): 6.55 (3.22–13.30)] and SDH [OR (CI_95%_): 4.56 (1.72–12.12)]. Further pathology comparisons for different road users can be found in the supplementary material (Supplementary Figs 1 and 2).

### Predicting moderate–severe TBI risk with delta-*V*

Our injury risk curves can be used to predict moderate–severe TBI risk for someone involved in a collision with a known delta-*V* value. Three distinct models to predict moderate TBI are shown with their corresponding ROC_AUC_ curves to evaluate performance (Fig. 8). For the combined pedestrian–cyclist group, moderate–severe TBI was differentiated from all other severities (Fig. 8A) and is a fair predictor of TBI severity [ROC_AUC_ (CI_95%_): 0.73 (0.55–0.89)] (Fig. 8D). The predictive capability for the model car occupant group to differentiate moderate–severe TBI from all other severities (Fig. 8B) is good [ROC_AUC_ (CI_95%_): 0.81 (0.68–0.93)] (Fig. 8C). When additionally including a flag for higher lateral delta-*V* component, this increases marginally [ROC_AUC_ (CI_95%_): 0.84 (0.75–0.91)] (Supplementary Fig. 3A). We finally compare a model which differentiates extremely well between car occupants with moderate–severe TBI and uninjured car occupants (Fig. 8C). It had a high TBI detection capability [ROC_AUC_ (CI_95%_): 0.93 (0.84–1.00)] (Fig. 8F) demonstrating excellent classification capability of uninjured and moderate–severe TBI groups in particular. This again increases further when considering the dominant delta-*V* component [ROC_AUC_ (CI_95%_): 0.95 (0.89–1.00)] (Supplementary Fig. 3B). Further information about the two moderate–severe TBI risk models with a baseline of all other severities (Fig. 8A and B) including precision and recall corresponding to different risk cut-off thresholds (5–50%) can be found in Supplementary Table 7.

## Discussion

There is a poor understanding of the relationship between collision dynamics and TBI pathology, despite an estimated 34 million people sustaining TBI in RTCs each year.^2^ This limits the ability to reduce the risk of significant TBI occurring. We investigated the interaction between collision dynamics, TBI pathology and vulnerability (type of road user) using data from the RAIDS database collected on behalf of the UK Government’s Department for Transport. Detailed collision and clinical data were analysed from more than 5000 subjects involved in RTCs. We described the prevalence of different types of TBI pathology and model the relationship of injuries to collision dynamics, characterized by estimated change in velocity of the vehicle or VRU during the impact phase of the collision (delta-*V*). We show that in cyclists, wearing a helmet is protective against TBI of all severities and moderate–severe TBI, particularly skull fracture and SDH and that this is not due to non-helmeted cyclists travelling faster or being impacted by vehicles travelling at higher speeds. Moderate–severe TBI risk increased with delta-*V* and was significantly higher in VRUs for a given delta-*V*. The data allowed us to estimate thresholds of delta-*V* for different types of TBI and highlighted the importance lateral delta-*V* has on increasing TBI risk in car occupants. The results have the potential to influence trauma care directly by informing the development of advanced automated collision notification (ACN) systems that are increasingly being fitted in new vehicles.

Clinical records and post-mortem reports provided information about the nature of TBI pathology of 5374 subjects involved in 2065 collisions. Five hundred and ninety-five subjects sustained a TBI (20.2% of 2940 casualties) of which the majority were moderate–severe (52.9% of 595 TBI subjects). SAH, focal brain injury, skull fracture and SDH were all common pathologies. As expected, the risk of moderate–severe TBI was significantly (6 times) higher for VRUs than for car occupants for a given delta-*V*. Pedestrians were most at risk, supporting previous findings.^4^ In general, as the protection level provided by personal protective equipment such as helmets or the vehicle structure itself is increased, the overall rate and severity of TBI decreased illustrating the importance of head protection for VRUs.^47^ Our results are similar to other in-depth European databases. For example, a high prevalence of focal brain injury and skull fractures in pedestrians has previously been shown in German and Dutch RTCs.^48,49^

Our analysis of cyclist collision dynamics and helmet usage provided novel insights into the protection provided by helmets. Previous work has shown that helmets protect from TBI of all severities in RTCs and are particularly protective of moderate–severe TBI including a skull fracture and SDH.^50^ Our results provide further evidence that this is the case and additionally show that this protective effect was not simply due to differences in the speed of the cyclist, e.g. non-helmeted cyclists travelling faster or differences in the speed of the vehicle impacting the helmeted and non-helmeted cyclists. We show that non-helmeted cyclists are at greater risk of skull fracture, which can be explained by higher linear acceleration and contact forces, both of which are reduced by helmets.^51^ We also showed an increased risk of SDH in non-helmeted cyclists, which may be related to rotational rather than linear acceleration, with relative skull-brain motion thought to be the key mechanism of injury.^52–56^ The observations in this study from real-world collision data highlight that existing helmets are effective at mitigating a significant portion of TBI sustained in RTCs. Emerging helmet technologies have been developed based on increased understanding of specific TBI pathology injury mechanisms (e.g. intracranial bleeding).^47,56^ These new technologies have been shown to be even more effective in mitigating rotational effects.^57^ Continued development could further improve the protection provided by helmets for a range of road users including cyclists, motorcyclists and micro-mobility users.

We address a key knowledge gap in the understanding of how collision dynamics relate to specific types of TBI pathology in varying types of road users. The information we provide is important because the risk of TBI pathologies such as SDH or focal brain injury is related to collision dynamics and interact with the extent of an individual’s protection from injury i.e. their vulnerability as a road user. A surprisingly small amount of work has addressed this problem previously. Two small studies (<60 subjects) have shown relationships between collision dynamics (including delta-*V*) and subdural and intraventricular haemorrhage.^21,22^ Our results significantly increase understanding in this area by providing a detailed characterization of injury risk for specific TBI pathologies in terms of collision dynamics and road user vulnerability.

Detailed reconstructions for the collisions in RAIDS provided an estimation of collision dynamics including delta-*V*. This quantifies the change in total impact velocity for the vehicles or pedestrians involved in collisions. We observed no moderate–severe TBI below a delta-*V* of 19 km/h for car occupants and 8 km/h for VRUs. Delta-*V* for VRUs is heavily dependent on the speed of the impacting vehicle. To consider a typical example collision between a pedestrian and a car, common characteristics of pedestrian collisions must first be explored. Pedestrians and cyclists are commonly injured in urban areas, which in the UK tend to have a 20 or 30 mph speed limit.^58^ In addition, STATS19 GB data during the period of our study showed that the majority (70%) of pedestrians involved in collisions were crossing a road at the time. Therefore we consider a typical example collision between a pedestrian crossing a road and a car travelling at 32 km/h (20 mph) at the point of impact. In this case, the pedestrian’s movement is perpendicular to the direction of travel of the impacting car. The pedestrian is accelerated to the speed of the car during impact, so the VRU delta-*V* may be considered to be equivalent to the car impact speed (32 km/h). In this scenario, the risk of moderate–severe TBI is 26% (CI_95%_: 24.7–27.7%). Alternatively, for the same collision configuration with the car instead of travelling at 48 km/h (30 mph) at the point of impact, the VRU delta-*V* is 48 km/h. In this higher delta-*V* scenario, the risk of moderate–severe TBI increased to 39% (CI_95%_: 36.5–43.5%). The risk of TBI pathologies approximately doubled from a 32 to 48 km/h delta-*V* (skull fracture: 18.3 versus 37.0%, subarachnoid: 17.9 versus 30.2% and focal: 18.2 versus 31.6%) with SDH risk remaining lower (9.2 versus 14.7%) at both delta-*V* points. These results cannot be directly extrapolated to the speed limits on roads because cars travel at a range of speeds and brake variably prior to and during impact. Further research could usefully explore the risks of TBI in specific speed zones.

We demonstrate for the first time that increasing delta-*V* had a distinct effect on the risk of different TBI pathologies for car occupants and VRUs. In VRUs, we showed that skull fracture risk increased particularly rapidly with increasing delta-*V* when compared with car occupants. For example, the risk of skull fracture for VRUs increased dramatically from 18% at 32 km/h delta-*V* to 37% at 48 km/h delta-*V* to 60% at 64 km/h delta-*V*. For car occupants, skull fracture risk was 2% at 32 km/h, 4% at 48 km/h and 9% at 64 km/h. Skull fractures have previously been shown to increase with increasing vehicle impact speed for pedestrians.^59^ High linear accelerations and direct head impacts are known to cause skull fractures.^60,61^ Hence, the difference in skull fracture risk is most likely to be because pedestrians and cyclists are at greater risk of direct head impacts that often result in skull fractures. In contrast, vehicle occupants are relatively protected from direct head impacts by the routine use of restraint systems within a vehicle.

We were also able to directly compare the TBI prevalence and risk associated with lateral and longitudinal delta-*V* for car occupants. Side impacts and rollover collisions tend to have high lateral delta-*V* components and have previously been linked to serious head injury.^14,62,63^ Our investigation of a large number of collisions allowed us to study collisions with only lateral or longitudinal delta-*V*, allowing the contribution of different delta-*V* directions to be studied more precisely. Lateral delta-*V* increased the risk of TBI and SAH compared with longitudinal delta-*V*, even when total delta-*V* was lower. The results suggest that future vehicle safety modifications designed to reduce car occupant TBI risk should focus on protecting from the effects of lateral vehicle delta-*V*. Potentially modifiable mechanisms include head contact with the internal side structures of the vehicle and high angular or rotational accelerations of the head, which can cause SAH.^64^ Overall risk prediction capability was also improved when including dominant lateral delta-*V* as a binary flag.

We selected logistic regression as an established and most widely used tool for constructing injury risk relationships, particularly in RTCs.^42–44^ Despite being a powerful tool capable of discerning the importance of parameters contributing to risk, some limitations arise. For example, the lack of representability within the RAIDS sample must be considered before assuming wider applicability of the risk functions derived for the RAIDS data. Owing to RAIDS being a serious subset of GB collisions, uninjured subjects and slightly injured casualties are underrepresented within the data relative to true national incidence rates. Under-sampling relative to the true incidence rate has been shown to overestimate the injury risk for a given exposure level.^65^ We expect this effect to be particularly pronounced in the risk functions for the pedestrian/cyclist group as they are most significantly underrepresented (as shown in Fig. 3), contributing to the non-zero risk observed at zero delta-*V* (Fig. 8). A similar risk overestimation effect is likely to present to a lesser degree in car occupants. Previous work has explored the effect of specific modelling choices on underreporting in specific RTC datasets.^66^ A range of alternate parametric modelling approaches to address different nuances of RTC data are discussed in detail by Savolainen *et al*.^43^ Without applying more complex modelling choices, there are alternate parametric models available which would tie the non-zero risk observed at zero delta-*V* in the RAIDS pedestrian/cyclist group to zero. For example, although not typical for larger datasets, applying a Weibull regression model could necessitate the expected zero risk at zero delta-*V* relationships, as demonstrated in this single-dependent variable example in a related field.^67^ Future work could usefully investigate the effect of different parametric modelling choices on risk functions derived from RAIDS data.

We reported the estimated prevalence of different types of TBI. Our estimate of moderate–severe TBI prevalence (53% of RAIDS TBI) was higher than the proportion of severe TBI previously observed in Trauma Audit Research Network data in England and Wales during our study period (46% of RTC TBI).^68^ RAIDS was designed to capture serious RTCs and are likely to underestimate the prevalence of mild TBI produced by less serious RTCs. We partially accounted for this by scaling the RAIDS estimates using information from the STATS19 database. This GB database includes all police-reported casualties and is the most comprehensive GB data available. However, police data also report only a subset of all road casualties, and up to 45% of RTC hospital admissions are omitted from STATS19. Collisions involving motorcyclists and particularly cyclists, as well as more minor injuries, are known to be underreported.^69–71^ Therefore, we expect the RR estimates we report for the cyclist and motorcyclist groups to underestimate the true RR. Nevertheless, our estimated rate of mild–probable and moderate–severe TBI in national police-reported collisions of 6% is similar to a previous study French study that estimated a rate of 6.7% for TBI following RTCs.^72^

DAI is an important TBI pathology commonly caused by RTCs.^73^ High shear forces produced at the time of RTCs cause damage to white matter tracts in the brain leading to DAI.^9,74^ Catastrophic outcomes after TBI such as persistent vegetative state are often caused by the presence of extensive DAI. Previous reports RTC databases have estimated relatively low rates of DAI, between 0.1 and 6.3%.^17,24,75^ We find a similarly low rate in the RAIDS data (4.4% of all TBI). However, it is likely that these rates are significantly underestimated which is a limitation in this study. The classifications of TBI pathology from clinical data in RAIDS and other databases of this type are based mainly on CT imaging, which often misses significant DAI.^76^ Advanced magnetic resonance imaging provides a more sensitive way of diagnosing DAI.^77^ Diffusion tensor imaging allows DAI to be identified in individuals and suggests around 50% of moderate–severe TBI have some degree of DAI.^78^ Radiology reports from magnetic resonance and diffusion tensor imaging within RAIDS are far less common that CT imaging or post-mortem reports, which limits our ability to fully diagnose DAI, particularly in surviving patients. In keeping with its adverse clinical effects, two-thirds of the patients with evidence of DAI in RAIDS died. This emphasizes the importance of considering the collision dynamics which might lead to DAI, as strategies to reduce the incidence of poor clinical outcomes after RTCs should focus on reducing the prevalence of DAI.

Our detailed investigation of the relationship between RTC dynamics and TBI is particularly timely because of the development of smart sensor technologies that are increasingly deployed in vehicles. These provide the information for ACN systems that detect collision events using event data recorders and can automatically notify emergency services of the exact collision location. The European ACN system, known as eCall, is now compulsory for all new cars, and has been shown to potentially reduce fatality rates by 5–10%.^79^ In US RTC data, if at least one vehicle involved has an ACN system, emergency service notification time is reduced (from median 4, interquartile range [IQR]: 2–9 min to median 2, IQR 1–5 min) and patients arrive at medical facilities faster, with particular benefits seen in less urban areas (median 36 versus 45 min).^80^ Advanced ACN systems can also provide emergency services with automated information about injury risk, which can enhance trauma care response further.^81–83^ In Europe in 2024, all new cars sold must record collision events in increased detail, including delta-*V* (lateral and longitudinal components).^84^

Trauma care is now generally concentrated within major trauma centres. Patients with serious TBI should be taken directly to a centre with neurosurgical capability. However, this does not always happen. Advanced ACN would be enhanced by the ability to predict the likelihood of life-threatening TBI. One British report found >50% of trauma patients requiring neurosurgical intervention were taken to hospitals without neurosurgical provisions and only 14% of TBI patients requiring hospital transfer were operated on within 4 h of injury.^85^ This is very problematic as delays in neurosurgery of this degree significantly impact clinical outcomes.^86,87^ In patients with severe TBI, mortality was reduced from 36 to 19% when transferring directly to a trauma centre with neurosurgical provision.^88^ Hence, improved clinical outcomes after RTC could be delivered by the automated identification of collisions with a high risk of producing serious TBI, as this alert could be used to divert patients directly to an appropriate major trauma centre. Our results (based on delta-*V*) could inform future TBI-specific advanced ACN systems.

The exceptionally detailed clinical and collision data enabled us to investigate the interaction between injury pattern (pathology), vulnerability (type of road user) and RTC dynamics (using delta-*V*) for the first time. The risk with increasing delta-*V* of sustaining moderate–severe TBI pathologies is higher for VRUs than car occupants, likely due to their decreased protection levels. Skull fracture risk in particular increases substantially with increasing delta-*V* for VRUs, which aligns with the known injury mechanism of high linear acceleration and contact force relating to speed. For car occupants, there is a higher risk of moderate–severe TBI in lateral delta-*V* only collisions than equivalent longitudinal delta-*V* collisions, particularly SAH. By basing our TBI risk analysis on delta-*V*, our work has the potential to impactfully inform real-world ACN systems that guide post-accident response providing that they can detect delta-*V* reliably.

## Supplementary Material

fcac033_Supplementary_Data

## Abbreviations

ACN =: automatic collision notification
AIS =: abbreviated injury scale
AUC =: area under the curve
CI =: confidence interval
DAI =: diffuse axonal injury
MW =: Mann–Whitney
RAIDS =: Road Accident In-Depth Studies
ROC =: receiver operator characteristic
RR =: relative risk
RTC =: road traffic collision
SAH =: subarachnoid haemorrhage
SDH =: subdural haematoma
TBI =: traumatic brain injury
TRL =: Transport Research Laboratory
VRU =: vulnerable road user
