# Supplementary material for: The relationship between road traffic collision dynamics and traumatic brain injury pathology
Source: Brain Commun. 2022 Feb 12;4(2):fcac033. doi: 10.1093/braincomms/fcac033 (PMC8914876; doi:10.1093/braincomms/fcac033)
Supplement: fcac033_Supplementary_Data [file fcac033_Supplementary_Data.pdf]

Supplementary Material

Supplementary Table 1: Collection criteria for RAIDS on-scene and retrospective data

Criteria	RAIDS Phase 1 (April 2013 – December 2015) – heavy vehicle	RAIDS Phase 1 (April 2013 – December 2015) - car	RAIDS Phase 2 (April 2016 – April 2020) - car
**Cases by injury severity**	No injury target	No injury target	60% KSI in sample vehicles
**Sample area**	All of Thames Valley and Hampshire (excluding Isle of Wight)	All of Thames Valley	All of Thames Valley and Hampshire (excluding Isle of Wight)
**Collisions must include**	At least one vehicle involved was a:<br>• heavy goods vehicle (HGV, GVW >3,500kg)<br>• light goods vehicle (LGV, GVW <3,500kg), including small vans and pick-ups that don't have passenger car equivalents<br>• large passenger vehicle (buses >16 passenger seats)<br>• minibus (8-16 passenger seats)<br>• Other motor vehicle (recovery vehicle, refuse collection vehicle etc.)	M1 vehicle that was seven years old or less at the time of the collision had at least one occupant who was injured (according to the initial police injury severity assessment)	M1 vehicle that was five years old or less at the time of the collision, and had at least one occupant who was injured (according to the initial police injury severity assessment) and required hospital treatment
**Vehicles for inspection**	The sample vehicle is available for subsequent inspection	Sample vehicle was towed and available for inspection	Sample vehicle was towed and available for inspection.<br>All towed vehicles must be examined
**Injury criteria for collision**	There is at least one injured road user in either the large vehicle or involved in the collision with the large vehicle		

Supplementary Table 2: Mayo TBI Classification System Search Terms

The terms shown in Supplementary Table 2 were used in the free-text search algorithm to extract TBI information from the RAIDS database. The terms were selected and categorised by Mayo TBI severity by the authors before being reviewed by an independent histopathologist and TBI clinician. The terms were refined using RAIDS Phase 1 and 2 data and validated manually for 507 subjects involved in 200 collisions from Phase 2 Extension data (2019-2020) obtaining ≥99.4% agreement. Our method also captured all AIS injury-coded pathologies it was possible to directly compare (subdural haematoma, subarachnoid haemorrhage and skull fracture). Sentences containing the terms were extracted for further analysis. Where terms related to false positives were found, for example 'no' preceding a TBI search term, these were flagged for manual review. Note that skull fracture-related search terms were put in the mild category, but the sentences they appeared in were assessed for severity. The vast majority of skull fractures which were classified as moderate-severe also presented with other moderate-severe TBI terms. Following false positive assessment, we reclassified our casualties to ensure that the maximum TBI severity was correct.

Mayo Severity	Search Terms
Moderate-Severe (n=329)	pupils restrict, pupil restrict, brain bleed, bruising to brain, intra cranial pressure, no pupillary reaction, contrecoup, contre-coup, contre coup, contra-coup , contracoup, contra coup, coup injury, coup brain injury, tonsillar herniation, downward cerebellar herniation, coning, bolt, anneurism, aneurism, anneurysm, aneurysm, pupils unreactive, pupil unreactive, unreactive pupil, non-reactive pupil, pupils non-reactive, pupil non-reactive, pupils not react, pupil not react, pupils fixed, fixed pupil, pupil fixed, pupil dilated, pupils dilated, dilated pupil, coma , neurorehab, neuro rehab, neuro-rehab, EVD , extra ventricular drain, external ventricular drain, ICP monitoring, cranial pressure, ICP, cranioplasty, bone flap, burr hole, cerebral sinus, venous sinus, callosal, craniotomy, craniectomy, brain stem, brainstem, brain-stem, cerebellum, cerebrospinal fluid, cerebral-spinal fluid, CSF, CTE, chronic traumatic encephalopathy, neurological, neurosurg, brain contusion, the dura , meninges, dural, arachnoid, CSF fistula, diffuse axonal injur, DAI , \(DAI\), epidural hematoma, epidural haemorrhage, epidural hemorrhage, epidural bleed, frontal lobe, intracerebral, intracranial hematoma, intracranial bleed, intracranial haemorrhage, intracranial hemorrhage, intracranial haematoma, intercranial hematoma, intercranial haematoma, intracerebral hematoma, intercranial bleed, intercranial haemorrhage, intercranial hemorrhage, sub dural, sub-dural, intra-cranial, sub-arachnoid, intra cranial, sub arachnoid, subdural hematoma, subdural haematoma, subdural bleed, subdural haemorrhage, subdural hemorrhage, subarachnoid hematoma, subarachnoid haematoma, subarachnoid bleed, subarachnoid

	haemorrhage, subarachnoid hemorrhage, occipital lobe, parietal lobe, penetrating brain injury, arachnoid membrane, temporal lobe, traumatic brain injury, grey matter, white matter, grey-white matter, white-grey matter, brain tissue, decompressive craniectomy, cerebral, extra axial hematoma, extra-axial hematoma, extra axial haematoma, extra-axial haematoma, extra axial bleed, extra-axial bleed, extra-axial haemorrhage, extra axial haemorrhage, extra-axial hemorrhage, extra axial hemorrhage, corpus callosum, microhaemorrage, microhaemorrhage, microhemorrage, microhemorrhage, posterior fossa, dura mater, pia mater, cortical contusion, cerebral cortex, midline shift, sub-frontal contusion, occipital contusion, occipital haematoma, occipital hematoma, occipital haemorrhage, occipital hemorrhage, cortical infarction, Cerebral infarction, cerebral laceration, intraparenchymal haemorrhage, intraparenchymal hemorrhage, intraparenchymal haematoma, intraparenchymal hematoma, interparenchymal haemorrhage, interparenchymal hemorrhage, interparenchymal haematoma, interparenchymal hematoma, midbrain, mid-brain, gyrus, gyri, sulcus, sulci, third ventricle, 3rd ventricle, fourth ventricle, 4th ventricle, cerebral aqueduct, hippocampus, lateral ventricle, thalamus, cerebral hemisphere, amygdala, limbic system, pituitary fossa, sella turcica, cranial nerve, Wernicke, Broca, bihemispheric, bi-hemispheric, TBI, \(TBI\), brain substance, brain laceration, brain showed contusion, cerebral hemisphere, intraventricular haemorrhage, intraventricular hemorrhage, intraventricular haematoma, intraventricular hematoma, intraventricular bleed, interventricular haemorrhage, interventricular hemorrhage, interventricular haematoma, interventricular hematoma, interventricular bleed, exposing the brain, exposed the brain, brain was exposed, fragmentation of the brain, thalamic parenchymal haematoma, evulsion of the brain, temporal pole contusion, pole contusion, bilateral brain, brain parenchyma, brain parnchyma, parenchymal contusion, parnchymal contusions, parenchymal contusions of the brain, parnchymal contusions of the brain, parenchymal brain contusions, parnchymal brain contusions, head injury - post traumatic punctate h, head injury - post-traumatic punctate h, temporal horn, quadrigeminal plate, brain bruis, bruises to the brain, bruise to the brain, bruising to the brain, bruises of the brain, bruise of the brain, bruising of the brain, Brain had been effectively eviscerated, Brain: small severely torn fragments, contusion to the inferior aspect of the brain, Haemosiderin deposition in the brain, pneumocephalus, brain was oedematous, displaced skull fracture, brain is swollen, brain was swollen, brain is diffusely swollen, brain was diffusely swollen, swollen brain, brain swelling, brain oedema, Oedema on the right side of brain, Oedema on the left side of brain, Oedema on the right side of the brain, Oedema on the left side of the brain, occipital condyle fracture, hemorrhagic contusion, haemorrhagic contusion, basilar skull fracture, intracranial abnormalit, intercranial, abnormal head CT, hydrocephaly, GCS3, GCS 3, GCS of 3, Glasgow Coma Score 3, GCS4, GCS 4, GCS of 4, Glasgow Coma Score 4, GCS5, GCS 5, GCS of 5, Glasgow Coma Score 5, GCS6, GCS 6, GCS of 6, Glasgow Coma Score 6, GCS7, GCS 7, GCs of 7, Glasgow Coma Score 7, GCS8, GCS 8, GCS of 8, Glasgow Coma Score 8, GCS9, GCS 9, GCS of 9, Glasgow Coma Score 9, GCS10, GCS 10,

	GCS of 10, Glasgow Coma Score 10, GCS11, GCS 11, GCS of 11, Glasgow Coma Score 11, GCS12, GCS 12, GCS of 12, Glasgow Coma Score 12, GCS: 3, Glasgow Coma Score: 3, GCS: 4, Glasgow Coma Score: 4, GCS: 5, Glasgow Coma Score: 5, GCS: 6, Glasgow Coma Score: 6, GCS: 7, Glasgow Coma Score: 7, GCS: 8, Glasgow Coma Score: 8, GCS: 9, Glasgow Coma Score: 9, GCS: 10, Glasgow Coma Score: 10, GCS: 11, Glasgow Coma Score: 11, GCS: 12, Glasgow Coma Score: 12, extra dural hem, extra-dural hem, extradural hem, extra dural haem, extra-dural haem, extradural haem, extra dural bleed, extra-dural bleed, extradural bleed, extra dural blood, extra-dural blood, extradural blood, extra dural, extra-dural, extradural, axonal , axonal inj
Mild-Probable (n=148)	GCS13, GCS 13, GCS of 13, Glasgow Coma Score 13, GCS14, GCS 14, GCS of 14, Glasgow Coma Score 14, GCS: 13, Glasgow Coma Score: 13, GCS: 14, Glasgow Coma Score: 14, concussive, basilar fracture, skull fracture, fracture of the skull, fractured skull, occipital fracture, occipital bone fracture, fracture of occipital bone, fracture the occipital bone, temporal fracture, temporal bone fracture, fracture of temporal bone, fracture the temporal bone, parietal fracture, parietal bone fracture, fracture of parietal bone, fracture the parietal bone, frontal fracture, frontal bone fracture, fracture of frontal bone, fracture the frontal bone, head fracture, maxilla fracture, maxilla bone fracture, fracture of maxilla, fracture the maxilla, depressed skull fracture, linear skull fracture, mandibula fracture, mandibula bone fracture, posterior fossa, ring fracture of the skull, forament magnum, foramen magnum, compressed skull fracture, skull showed a fracture, skull showed a complex fracture, skull base showed fracture, skull showed fracture, skull was fractured, base of skull fracture, post-traumatic amnesia, post-traumatic anterograde amnesia, post-traumatic retrograde amnesia, PTA, subgaleal haematoma, subgaleal hematoma, subgaleal haemorrhage, subgaleal hemorrhage, amnesia, post-traumatic amnesia, post-concussion syndrome, concussion, loss of consciousness, ko'd, K/O, ko'ed, k/o'd, knocked out, Unconscious, not conscious, unresponsive, fracturing of the sphenoid, fractures to the skull vault, parietal bone, sphenoid bone, fractures to the skull base, skull showed fracture, fracture to the left occipital, fracturing of the ethmoid, fracture to the occipital, roof of the left orbit, sphenoid fracture, fracturing of the frontal plates, fractures of vault, ethmoid, frontal bone, fractures of the vault, skull showed extensive fractures, cranial fossa, fracture of the right temporal bone, fracture of occipital condyle, temporal bone, fractures of the base of skull, occipital condyle fracture, temporal bone was fractured, basiocciput, fracture of right occipital condyle, skull bones showed multiple complicated fractures, skull showed extensive comminuted, ethmoid bone, fracture of the temporal bone, skull base extension, fractures of the anterior fossa, petromastoid fracture, skull base showed multiple comminuted fractures, sphenoid, skull bones, roof of the right orbit, base skull with a fracture, fractures of skull vault, superomedial orbit, skull bones show multiple, occipital bone, undisplaced occiput fracture, skull base missing, occiput fracture, base of skull fracture, comminuted fracture of the vault, frontal fracture, fracture base of skull, skull showed extensive fracture, fracture through the left orbit, severe injury of the head mainly on left side with comminuted fracture of bone,

	fracture of left occipital condyle, fractures of skull base, fractures to the vault, fracture at the base of skull, skull - basilar fracture, skull - vault fracture, fractures of the skull, fractured base of skull, skull base fracture, skull vault fracture, fracture - vault of skull, fracture - base of skull, fracture of the left temporal bone, skull shows fractures, fracture to the right occipital, skull fracture, fracture of the frontal bone, fracture of skull, fracture of base of skull, temporal fracture, skull showed extensive, fractures of the skull vault
Symptomatic-Possible (n=27)	blurred vision, blurry vision, blurred sight, blurry sight, double vision, confusion, confused, daze, dazed, dizziness, dizzy, focal neurologic symptoms, focal neuro, headache, head ache, nausea, nauseous, vomit, vomiting, seizure, head pain, agitated, agitation, queezy, feel sick, felt sick, feeling sick
Negative (Exclusion) (n=24)	past history, past medical history, medical history, pre existing condition, existing condition, pre-existing, pre existing, preexisting, image quality , artefact, GP , general practitioner, underlying medical conditions, intact, not, no, within normal limits, normal , no evidence of , hypoxic brain injury, lung*, overlaying **, over the **<br><br>** relating to focal contusion*<br>*** skull fracture terms*

Supplementary material is continued on the next page.

Detailed Description of Method for Scaling Findings to GB Level

The national level STATS19 data is collated by the police. STATS19 includes information about the type of road user involved in the collision, their overall injury severity, details about the road a collision occurs on, environmental factors such as lighting and collision causation. Collisions must be attended by the police or be reported to the police within 30 days. Not all collisions are reported to the police, with those causing minor injuries more likely to be missed. STATS19 data does not report injury pathology. Collisions involving cyclists are particularly known to be underrepresented (*47*). RAIDS is a subset of STATS19 that focusses on severe injuries and fatalities, whereas STATS19 covers injuries of all severities. A mapping is therefore required to scale RAIDS results to STATS19. We use fields available both in RAIDS and STATS19 to create a mapping, following similar methodology to other in-depth database scaling, refined by TRL statisticians to best encompass the GB scenario[30].

Seven collision characteristics present in both datasets were considered for relating the RAIDS and STATS19 populations. The shortlisted collision variables were road user type, casualty age, lighting level, speed limit, road class and vehicle age and overall injury severity (as an output). These were chosen because they are the most important to the GB scenario and relate to the selection criteria for RAIDS cases. For each group in RAIDS and STATS19, we counted the number of casualties who met a given combination of the six input collision characteristics. The counts for a given combination of collision characteristics were compared in corresponding RAIDS and STATS19 populations. We applied chi-squared tests to ensure the casualty counts for our chosen scaling variables in RAIDS and STATS19 were significantly different. We used R to fit decision tree models with injury severity as the outcome variable to determine which input variables were most important in classifying RAIDS by injury severity. This analysis was performed with a minimum cluster size of 49 and a maximum depth of 2 variables. We applied chi-squared tests to ensure the casualty counts for our chosen scaling variables differed significantly in RAIDS and STATS19.

As the collection criteria for RAIDS was slightly different in each phase, we first split the data into a Phase 1 group and a Phase 2 subset. Each phase of RAIDS data is then further split into two subsets depending on whether investigators attended the scene. As each of these subsets have different selection criteria, we applied the same selection criteria to split the STATS19 data into four subsets: Phase 1 On-Scene, Phase 1 Retrospective, Phase 2 On-Scene and Phase 2 Retrospective. For each of these four subsets, a decision tree analysis was performed to select

the scaling variables. A weighting is then calculated for casualties who are grouped by the chosen combination of collision factors. Overall injury severity and road user type were selected to determine the weighting for casualties involved in all on-scene cases. In addition to road user type and overall injury severity, vehicle age was also included to calculate weightings for casualties involved in Phase 1 Retrospective cases. Overall injury severity, road class and vehicle age were used to calculate weightings for casualties involved in Phase 2 Retrospective cases. The weighting value, $\boldsymbol{W}$, is calculated from the normalised ratio of S19 casualties divided by the normalised ratio of RAIDS casualties $\boldsymbol{R_{N,\,S19}/R_{N,\,RAIDS}}$ where the normalised ratio for each group is given by the casualty numbers of a given factor combination divided by all casualties in the subset where $\boldsymbol{R_N} = \frac{\boldsymbol{casualty\ count\ for\ given\ factor\ combination}}{\boldsymbol{casualty\ count\ for\ all\ factor\ combinations\ in\ subset}}$. These weights are then applied in subsequent analysis to calculate casualty numbers at GB level when the information of interest, such as TBI severity, is only available at RAIDS level.

Weights for RAIDS Phase 1 and Phase 2 casualties are shown below. Generally, slightly injured casualties have higher weights (>1) as they are underrepresented in RAIDS, while seriously or fatally injured casualties have lower weights (<1) as they are overrepresented.

Supplementary Table 3: Phase 1 On-Scene Investigation Weights

Overall Injury Severity	Road User Type	Weight
Slight	Pedestrian	7.355830
Slight	Cyclist	6.824997
Slight	Motorcyclist	3.652496
Slight	Bus occupant	2.816873
Slight	Light goods vehicle occupant	2.643330
Slight	Car occupant	2.572593
Serious	Cyclist	2.178748
Slight	Minibus occupant	1.823748
Serious	Other	1.771199
Slight	Heavy goods vehicle occupant	1.566671
Serious	Motorcyclist	1.103831
Serious	Pedestrian	1.018075
Serious	Car occupant	0.872733
Serious	Heavy goods vehicle occupant	0.687769
Fatal	Pedestrian	0.601219
Serious	Light goods vehicle occupant	0.446884
Fatal	Motorcyclist	0.372477
Fatal	Car occupant	0.370760
Serious	Minibus occupant	0.318383
Fatal	Cyclist	0.302928

Supplementary Table 4: Phase 1 Retrospective Investigation Weights

Overall Severity	Road User Type	Casualty Age	Weight
Slight	Bus occupant	65+ years	18.01404
Slight	Car occupant	0-15 years	4.269710
Slight	Bus occupant	45-64 years	4.210342
Slight	Motorcyclist	16-24 years	4.101608
Slight	Car occupant	45-64 years	3.207763
Serious	Motorcyclist	16-24 years	3.061066
Slight	Car occupant	16-24 years	3.009888
Slight	Car occupant	25-44 years	3.000169
Serious	Pedestrian	45-64 years	2.422426
Slight	Minibus occupant	25-44 years	2.273777
Slight	Light goods vehicle occupant	65+ years	2.147150
Slight	Car occupant	65+ years	1.970218
Serious	Motorcyclist	25-44 years	1.882886
Slight	Car occupant	Unknown	1.562954
Slight	Other	25-44 years	1.480983
Slight	Other	45-64 years	1.343345
Slight	Bus occupant	25-44 years	1.313816
Serious	Pedestrian	65+ years	1.244246
Slight	Bus occupant	16-24 years	1.214516
Serious	Motorcyclist	45-64 years	0.946948
Serious	Pedestrian	25-44 years	0.945113
Serious	Cyclist	25-44 years	0.921256
Slight	Light goods vehicle occupant	Unknown	0.886388
Slight	Light goods vehicle occupant	25-44 years	0.846545
Slight	Light goods vehicle occupant	45-64 years	0.793014
Fatal	Pedestrian	65+ years	0.754255
Serious	Car occupant	Unknown	0.704706
Slight	Light goods vehicle occupant	16-24 years	0.694088
Serious	Bus occupant	25-44 years	0.644145
Slight	Heavy goods vehicle occupant	25-44 years	0.591384
Fatal	Motorcyclist	45-64 years	0.545046
Slight	Other	16-24 years	0.545046
Fatal	Pedestrian	45-64 years	0.523024
Slight	Heavy goods vehicle occupant	45-64 years	0.485518
Serious	Cyclist	16-24 years	0.478980
Serious	Car occupant	0-15 years	0.348071
Serious	Car occupant	25-44 years	0.320467
Serious	Cyclist	65+ years	0.319320
Serious	Car occupant	16-24 years	0.304027
Slight	Light goods vehicle occupant	0-15 years	0.290416
Serious	Car occupant	65+ years	0.274889
Serious	Car occupant	45-64 years	0.267746

Slight	Heavy goods vehicle occupant	65+ years	0.258759
Serious	Other	45-64 years	0.236737
Fatal	Car occupant	16-24 years	0.228086
Serious	Light goods vehicle occupant	45-64 years	0.222973
Serious	Light goods vehicle occupant	16-24 years	0.206457
Serious	Light goods vehicle occupant	25-44 years	0.192269
Serious	Other	25-44 years	0.187187
Serious	Heavy goods vehicle occupant	25-44 years	0.171772
Fatal	Cyclist	25-44 years	0.154154
Fatal	Light goods vehicle occupant	45-64 years	0.143143
Serious	Heavy goods vehicle occupant	65+ years	0.143143
Serious	Bus occupant	16-24 years	0.137638
Serious	Heavy goods vehicle occupant	45-64 years	0.127728
Fatal	Motorcyclist	16-24 years	0.108275
Fatal	Car occupant	45-64 years	0.103504
Fatal	Car occupant	25-44 years	0.095043
Fatal	Car occupant	65+ years	0.087139
Fatal	Car occupant	0-15 years	0.085335
Serious	Minibus occupant	16-24 years	0.071572
Fatal	Heavy goods vehicle occupant	45-64 years	0.064231
Serious	Light goods vehicle occupant	65+ years	0.062763
Fatal	Cyclist	45-64 years	0.060561
Fatal	Heavy goods vehicle occupant	25-44 years	0.057808
Fatal	Light goods vehicle occupant	25-44 years	0.057808
Fatal	Light goods vehicle occupant	65+ years	0.055055
Fatal	Cyclist	16-24 years	0.030280
Fatal	Heavy goods vehicle occupant	65+ years	0.016517
Serious	Heavy goods vehicle occupant	Unknown	0.016517
Fatal	Light goods vehicle occupant	0-15 years	0.005506

Supplementary Table 5: Phase 2 On-Scene Investigation Weights

Overall Severity	Road User Type	Weight
Slight	Cyclist	8.841718
Slight	Pedestrian	8.534807
Slight	Other	3.706753
Serious	Light goods vehicle occupant	3.530349
Slight	Bus occupant	3.191199
Slight	Motorcyclist	3.161002
Slight	Car occupant	2.515547
Slight	Heavy goods vehicle occupant	1.555197
Serious	Pedestrian	1.355262
Serious	Bus occupant	1.220030
Slight	Light goods vehicle occupant	1.103440
Serious	Car occupant	1.087589
Serious	Cyclist	0.970018

Serious	Motorcyclist	0.555668
Fatal	Pedestrian	0.421598
Serious	Heavy goods vehicle occupant	0.419575
Fatal	Car occupant	0.229978
Fatal	Light goods vehicle occupant	0.172989
Fatal	Heavy goods vehicle occupant	0.150228
Fatal	Motorcyclist	0.100396
Fatal	Cyclist	0.091553

Supplementary Table 6: Phase 2 Retrospective Investigation Weights

Overall Severity	Road User Type	Casualty Age	Weight
Slight	Car occupant	25-44 years	2.806751
Slight	Car occupant	45-64 years	2.754580
Slight	Light goods vehicle occupant	45-64 years	2.643788
Slight	Car occupant	0-15 years	2.359920
Slight	Car occupant	16-24 years	2.042819
Slight	Light goods vehicle occupant	25-44 years	1.905829
Slight	Car occupant	65+ years	1.883100
Slight	Car occupant	Unknown	1.536850
Slight	Heavy goods vehicle occupant	25-44 years	0.565317
Slight	Other	25-44 years	0.460378
Serious	Motorcyclist	16-24 years	0.423141
Serious	Car occupant	65+ years	0.411858
Serious	Car occupant	25-44 years	0.388660
Serious	Car occupant	16-24 years	0.343209
Serious	Car occupant	0-15 years	0.317235
Serious	Light goods vehicle occupant	45-64 years	0.314817
Serious	Car occupant	45-64 years	0.280049
Serious	Car occupant	Unknown	0.262348
Slight	Light goods vehicle occupant	65+ years	0.243729
Fatal	Car occupant	45-64 years	0.209878
Slight	Heavy goods vehicle occupant	45-64 years	0.201980
Serious	Light goods vehicle occupant	25-44 years	0.189567
Fatal	Car occupant	65+ years	0.181951
Slight	Minibus occupant	45-64 years	0.152331
Fatal	Car occupant	25-44 years	0.129481
Fatal	Car occupant	16-24 years	0.086321
Slight	Light goods vehicle occupant	Unknown	0.077858
Serious	Light goods vehicle occupant	65+ years	0.054162
Slight	Minibus occupant	65+ years	0.037236
Slight	Minibus occupant	0-15 years	0.012694
Slight	Car occupant	25-44 years	2.806751
Slight	Car occupant	45-64 years	2.754580
Slight	Light goods vehicle occupant	45-64 years	2.643788

Slight	Car occupant	0-15 years	2.359920
Slight	Car occupant	16-24 years	2.042819
Slight	Light goods vehicle occupant	25-44 years	1.905829
Slight	Car occupant	65+ years	1.883100
Slight	Car occupant	Unknown	1.536850
Slight	Heavy goods vehicle occupant	25-44 years	0.565317
Slight	Other	25-44 years	0.460378
Serious	Motorcyclist	16-24 years	0.423141
Serious	Car occupant	65+ years	0.411858
Serious	Car occupant	25-44 years	0.388660

Supplementary material is continued on the next page.

Detailed Description of Delta-V Calculation

Vehicle delta-V is determined from crush profiles and initial trajectories where these are available (Fig. 2A). Vehicle crush measures are taken at the scene by expert collision investigators for all vehicles. Vehicle trajectories are estimated from physical evidence (e.g. skid marks or CCTV footage). The AiDamage program is used to reconstruct the collision from this information[34]. The CRASH3 algorithm is used to determine energy-related parameters including delta-V[33]. Longitudinal (front-to-back), lateral (side-to-side) and total delta-V are calculated for each vehicle. Vehicles generally have different delta-V value ($\Delta \boldsymbol{V_{V1}}$ and $\Delta \boldsymbol{V_{V2}}$ in Fig. 2Aii). Total delta-V comprises the Pythagorean sum of the longitudinal and lateral components, $\Delta \boldsymbol{V_{total}} = \sqrt{\Delta \boldsymbol{V_{longitudinal}}^2 + \Delta \boldsymbol{V_{lateral}}^2}$, capturing the directional transfer of force during the collision. If two vehicles are involved, crush measurements were required from both vehicles to calculate valid delta-V values. All car occupants with valid delta-V estimates from single impact phases were included. Where multiple impacts were present, delta-V was included only if one of the impact phases was clearly the injury-causing phase.

For VRUs, a hybrid approach is adopted. Pedestrian delta-V is approximated as the impact speed of the vehicle because most pedestrians in RAIDS had no velocity component in the direction the impacting vehicle was travelling as they are most commonly injured while crossing. Cyclists travel at higher speeds, sharing the carriageway with vehicles. Therefore, their initial speed can be influential on the delta-V and must be taken into account by combining the velocity of the cyclist with the impact speed of the vehicle involved in the collision (Fig. 2B). Vehicle impact speed at the start of the impact is determined using a combination of physical evidence such as CCTV or dashcam footage and physical evidence such as skid marks. In each collision configuration, the relative velocity is taken to account for the pre-crash directions of the VRU relative to the vehicle. For head-on collisions, the initial VRU speed is added to the impact speed of the other vehicle involved. In collisions where both the VRU and other vehicle involved have the same direction of travel, VRU speed was subtracted from the other vehicle's speed. Only the velocity component parallel to the direction of travel of the vehicle was considered ($\Delta V_{VRU} = V_{car\ initial} + V_{VRU\ initial}$). VRUs who were runover without being accelerated to the speed of the vehicle, for example those already lying in the road prior to impact, as the assumption that the VRU is accelerated to the speed of the impacting vehicle is not upheld and therefore cannot be used to calculate delta-V.

It is important to note that the delta-V we define refer to the change in velocity of the overall vehicle or VRU system during the injury causing impact phase and does not capture the specific delta-V of individual local regions. For car occupants, even while belted, the occupant's head is not perfectly coupled to the vehicle. The delta-V of local body regions may vary based on the kinematics of the VRU impact (i.e. the head may be accelerated towards the windscreen in certain collisions scenarios).

Supplementary material is continued on the next page.

Supplementary Figure 1: Logistic regression curves for distinct TBI pathologies

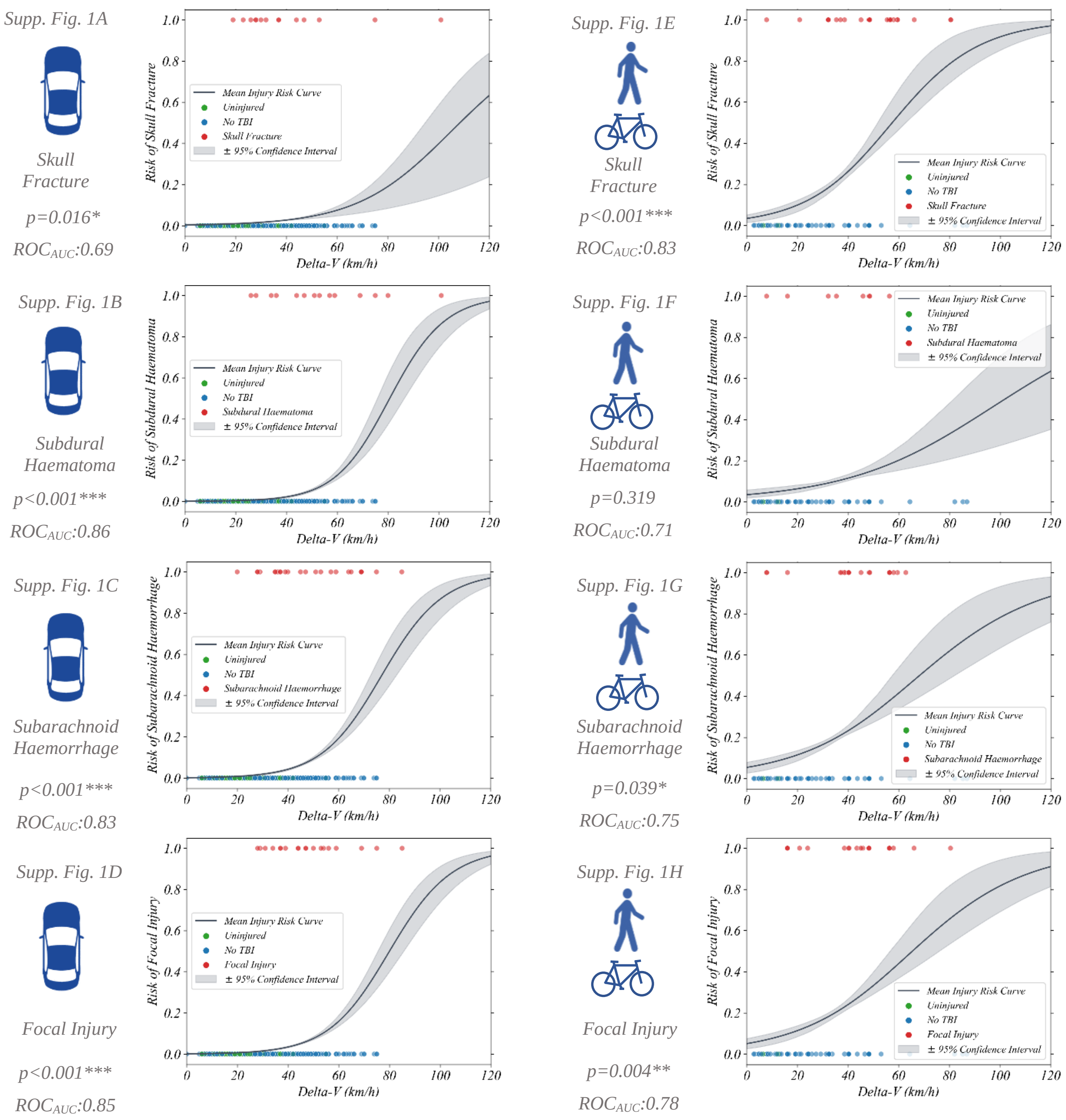

Supplementary Figure 1. Logistic regression models predicting the risk of sustaining TBI pathology from total delta-V (km/h). There are 651 car occupants without TBI in the baseline group (a-d), compared to 14 with skull fracture (A), 14 with subdural haematoma (B), 24 with subarachnoid haemorrhage (C) and 19 casualties with focal injury. For VRUs, there were 82 baseline casualties without TBI compared to 25 with skull fracture (E), 9 with subdural haematoma (F), 19 with subarachnoid haemorrhage (G) and 21 with focal injury (H). p-values indicate that delta-V is a significant predictive parameter for all instances except subdural haematoma in the pedestrian-cyclist group (possibly due to small sample size).

Supplementary Figure 2: Comparison of TBI pathology risk for different road users

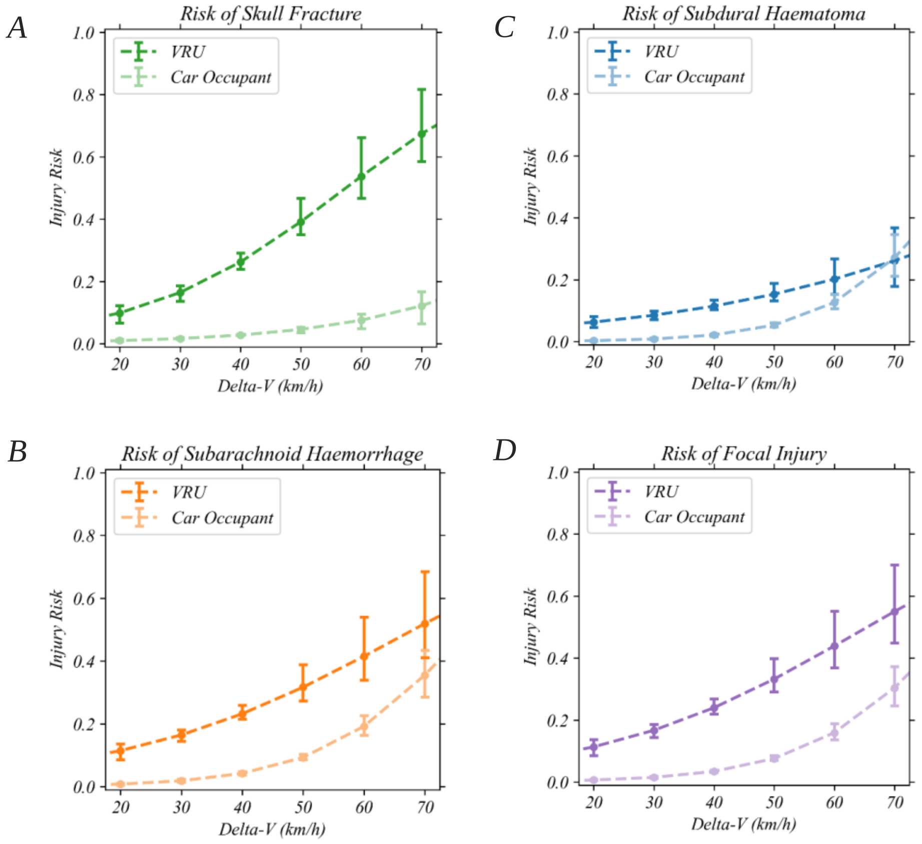

Supplementary Figure 2. Injury risk for different pathologies and road users. For 60km/h and below, the risks are significantly different for the car occupant and the VRU group

Supplementary Figure 3: Multivariate logistic regression for car occupant TBI risk

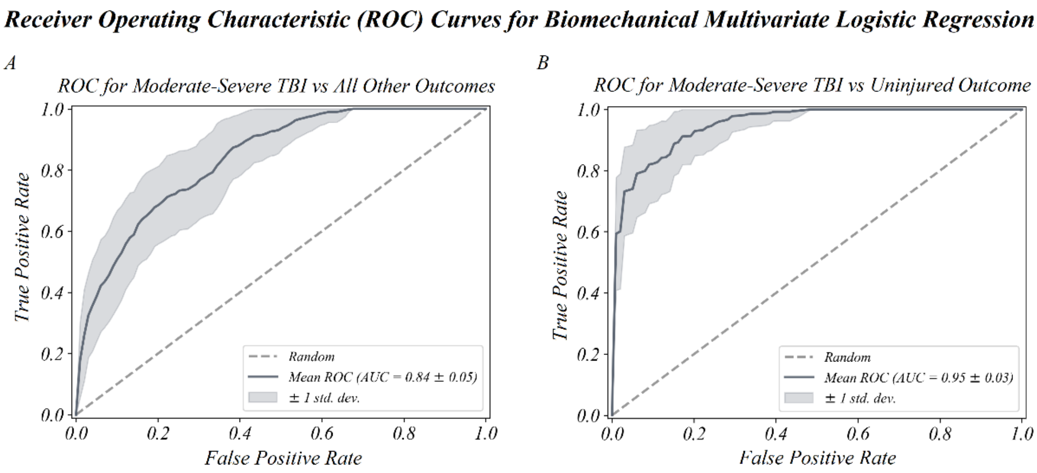

Supplementary Figure 3. ROC curves for car occupant risk of moderate severe TBI with baselines of (A) all other outcomes and (B) the uninjured cohort, constructed using multivariate logistic regression. Including the additional flag of dominant lateral delta-V increased the prediction capability of the models fitted in all instances (including pathologies).

Statistical Summary for Logistic Key Regression Models

To create the logistic regression models, the data was randomly split into k approximately equal-sized subsets. Stratification was used to ensure that in each subset the proportion of baseline and injury groups were representative of the overall dataset. Binary logistic regression models were trained on all data except k^{th} subset, which was withheld for testing. k-fold cross-validation was repeated 200 times with prespecified data seeds used to ensure repeatability when randomly shuffling the data prior to partitioning at the start of each iteration. Results from all 1000 iterations were recorded. The average injury risk curve was given by the 50th percentile of the ranked risk value at each point. 95% confidence intervals are again determined by taking the 2.5^{th} and 97.5^{th} ranked values at each point. To determine the predictive capability of our injury risk curves, we use the Receiver Operator Characteristic (ROC) curve and associated Area Under Curve (AUC) averaged over all 1000 iterations. We provide the precision (the number of correctly labelled positives divided by all labelled positives) and recall (the number of labelled positives divided by actual positives, also known as sensitivity) in the table below as these may be of particular interest for the application of these results to an Advanced ACN-type algorithm. Further work is required to ensure these results are generalisable and implementable to advanced ACN algorithms. For example, small differences (mean absolute error -4km/h) exist between CRASH3 and EDR delta-V in European vehicles (Lenard, et al., 2000), further research could usefully determine the current difference. Similarly, additional analysis and consideration of cut-off thresholds to ensure appropriate under- and over-triage rates are necessary prior to any real-world application.

Supplementary Table 7: Moderate-severe TBI logistic regression risk curve parameters

Road User Group	Severity Groups	LR Coeff. $[CI_{95\%}]$	ROC_{AUC} $[CI_{95\%}]$	Cutoff Threshold	Accuracy	Precision	Sensitivity / Recall (TPR)
Car Occupants	Moderate Severe vs baseline All Other	0.078 [0.053 - 0.103]	0.81 [0.68-0.93]	5%	0.745	0.125	0.641
				10%	0.875	0.209	0.480
				20%	0.937	0.397	0.300
				30%	0.945	0.484	0.243
				40%	0.948	0.548	0.163
				50%	0.949	0.638	0.127
Combined VRUs (Pedestrian and Cyclists)	Moderate Severe vs baseline All Other	0.0344 [0.013 - 0.056]	0.73 [0.55-0.89]	5%	0.296	-	-
				10%	0.305	0.308	0.964
				20%	0.545	0.380	0.831
				30%	0.690	0.489	0.712
				40%	0.712	0.563	0.375
				50%	0.703	0.518	0.173
